# Design Optimization of a Novel Catalytic Approach for Transglucosylated Isomaltooligosaccharides into Dietary Polyols Structures by *Leuconostoc mesenteroides* Dextransucrase

**DOI:** 10.1021/acs.jafc.4c04222

**Published:** 2024-09-18

**Authors:** Ana Muñoz-Labrador, Elisa G. Doyagüez, Silvana Azcarate, Cristina Julio-Gonzalez, Daniela Barile, F. Javier Moreno, Oswaldo Hernandez-Hernandez

**Affiliations:** † Institute of Food Science Research, CIAL (CSIC-UAM), Nicolás Cabrera 9, 28049 Madrid, Spain; ‡ Department of Food Science and Technology, University of California Davis, Davis, California 95616, United States; § Centro de Química Orgánica “Lora Tamayo” (CSIC), Juan de la Cierva 3, 28006 Madrid, Spain; ∥ Consejo Nacional de Investigaciones Científicas y Técnicas (CONICET), Godoy Cruz 2290 CABA (C1425FQB), 1033 Buenos Aires, Argentina

**Keywords:** sweetener, prebiotic, gluco-oligosaccharides, isomalto-oligosaccharides, acceptor reaction, glucosylation

## Abstract

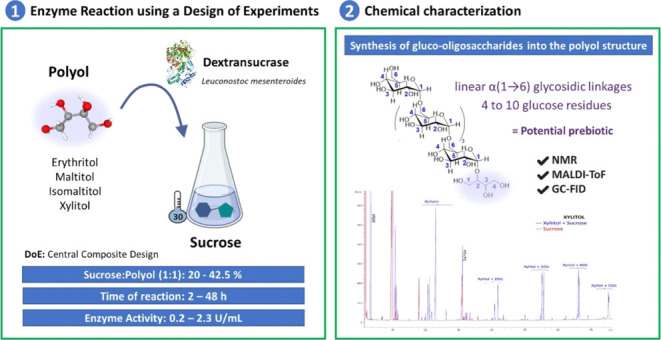

Polyols, or sugar alcohols, are widely used in the industry as sweeteners and food formulation ingredients, aiming to combat the incidence of diet-related Non-Communicable Diseases. Given the attractive use of Generally Regarded As Safe (GRAS) enzymes in both academia and industry, this study reports on an optimized process to achieve polyols transglucosylation using a dextransucrase enzyme derived from *Leuconostoc mesenteroides*. These enzyme modifications could lead to the creation of a new generation of glucosylated polyols with isomalto-oligosaccharides (IMOS) structures, potentially offering added functionalities such as prebiotic effects. These reactions were guided by a design of experiment framework, aimed at maximizing the yields of potential new sweeteners. Under the optimized conditions, dextransucrase first cleared the glycosidic bond of sucrose, releasing fructose with the formation of an enzyme-glucosyl covalent intermediate complex. Then, the acceptor substrate (i.e., polyols) is bound to the enzyme-glucosyl intermediate, resulting in the transfer of glucosyl unit to the tested polyols. Structural insights into the reaction products were obtained through nuclear maneic resonance (NMR) and matrix-assisted laser desorption/ionization time-of-flight (MALDI-TOF) analyses, which revealed the presence of linear α(1 → 6) glycosidic linkages attached to the polyols, yielding oligosaccharide structures containing from 4 to 10 glucose residues. These new polyols-based oligosaccharides hold promise as innovative prebiotic sweeteners, potentially offering valuable health benefits.

## 1 Introduction

In recent years, the increasing prevalence of obesity and related metabolic disorders has raised concerns about excessive consumption of traditional caloric sweeteners, such as sucrose. This has driven the search for healthier alternatives, leading to a surge in the development and utilization of Non-Sugar Sweeteners (NSS) in the food industry. NSS, encompassing both synthetic and naturally occurring or modified NNS, serve as low-/no-calorie alternatives to sugars. These NSS have emerged as candidates for sugar replacement, aiming to control blood glucose levels and prevent rising the prevalence of noncommunicable diseases (NCDs).^1^

To establish safety levels of intake (e.g., Acceptable Daily Intake, ADI), NSS undergo toxicological assessments. However, consensus on their long-term effectiveness remains elusive. A recent WHO guideline, based on a systematic review and meta-analysis, recommends not to use NSS for body weight and NCDs control.^2^ This recommendation does not apply to low-calorie sugars and polyols, which are sugars or sugar derivatives containing calories and are, therefore, not considered NSS.

Polyols, and more specifically sugar alcohols, so-called because they contain many hydroxyl groups, have gained prominence as promising sugar substitutes due to their unique properties and potential health benefits.^3^ Catalytic approaches are commonly used to produce polyols in the industry.^4^ They find wide application in various food and beverage products, pharmaceutical formulations, and oral care products while being considered Generally Regarded As Safe (GRAS). Commonly used polyols include sorbitol (E-420), xylitol (E-967), erythritol (E-968), and mannitol (E-965).^5,6^ These polyols offer a sweet taste akin to sugar and, unlike NSS they can function as bulking agents.^7,8^ Polyols have demonstrated health benefits; for instance, they undergo insulin-independent metabolism, preventing significant fluctuations in blood glucose levels,^4^ rendering them suitable for diabetic patients. They are also poorly digested in the gastrointestinal tract, although their rate of digestion and absorption varies among individual polyols.^9^ A portion of unabsorbed polyols serves as a substrate for bacterial fermentation in the large intestine, offering prebiotic effects.^10^ However, daily intake recommendations exist due to dose-dependent symptoms such as flatulence, distension, and laxative effects when consuming large quantities of polyols (>0.17–0.8 g/kg body weight).^11−13^

Polyols are naturally present in small amounts in several vegetable sources^14^ and are presently produced commercially using chemical hydrogenation of sugars. Consequently, biotechnological production of polyols offers a viable and sustainable solution to meet the demand.^4^ The prospect of biotechnological polyol production has spurred significant research. Within this context, biotechnological approaches for generating polyol-based sweetening derivatives present both challenges and allure.^15,16^

According to the CAZY classification, glucosyltransferases (EC 2.4.1.5) are members of the GH70 family, primarily produced by lactic acid bacteria.^17^ These enzymes, commonly called dextransucrases, are produced by the species of *Leuconostoc* and *Streptococcus* and catalyze the hydrolysis of the glycosidic bond in sucrose, releasing an enzyme-glucosyl covalent intermediate complex along with fructose.^18,19^ However, in the presence of a suitable acceptor molecule, dextransucrase can transfer glucosyl moieties to the acceptor, resulting in the formation of oligosaccharides with a degree of polymerization ranging from 2 to 10.^20^ Depending on the product they synthesize the main α-glycosidic linkages formed are α(1 → 3,4,6).^21^ Dextransucrases are well-characterized enzymes often used on an industrial scale to produce dextran polymer and oligosaccharides.^19^ By these means, it is possible to synthesize various types of oligosaccharides or glucoconjugates,^22^ Recent works have employed enzymatic modifications with natural sweeteners other than polyols,^23,24^ particularly dextransucrases, as seen in the study by Kang et al., which synthesized a rebaudioside-A-like compound.^25^

On the other hand, one-third of polyols that are consumed in the human diet are absorbed in the small intestine, although the amount of absorption varies depending on the individual polyol.^26^ Thus, low molecular weight polyols like erythritol and xylitol were reported to have small intestinal absorption rates of up to 90–95%.^12,27^ In this context, the production of glycosyl derivatives of polyols can be a good approach for broadening the number of microbiota-accessible carbohydrates (or derivatives) since these novel compounds could overlook the absorption at the level of the small intestine, becoming available for consumption by the colonic microbiota. Thus, the enzymatic production of β-monogalactosylated derivatives of xylitol^28,29^ has shown to be an efficient approach to developing novel prebiotic formulations by promoting the growth of beneficial gut commensal bacteria.

Enzymatic applications are well-known for producing Non-Digestible Oligosaccharides (NDOs) with prebiotic properties. The two most important commercially available NDOs are fructooligosaccharides and galactooligosaccharides (FOS and GOS).^30^ Previous studies have highlighted the prebiotic potential in oligosaccharides composed of glucose building blocks joined by linkages, at least, partially or slowly digestible to humans, namely gluco-oligosaccharides (GlcOS), such as isomalto-oligosaccharides (IMOS), oligodextran, nigero-oligosaccharides (GnOS), and Polydextrose.^31−34^

While Zhang et al., performed glycosylation on polyols, only erythritol was included as a sugar-polyol, resulting in a catalytic product with only one glucose residue attached to the polyol. This study strategically focuses on producing GlcOS with polyols as acceptors, yielding a novel class of sugar-free sweeteners with potential prebiotic properties and overcoming some of the drawbacks described for polyols.^35^

## 2 Materials and Methods

### 2.1 Chemicals

Four polyols were incorporated into the design of experiments (DOE) for screening and optimization purposes: xylitol, erythritol, maltitol, and isomaltitol, all of which were obtained from Carbosynth (Berkshire, U.K.). Dextransucrase from *L. mesenteroides* B512F was procured from CRITT Bio-Industries (Toulouse, France). Carbohydrate standards were sourced from Sigma-Aldrich (Madrid, Spain). All other chemicals utilized in the analysis were of analytical grade.

### 2.2 Experimental Design of Transglucosylation of Polyols

Enzymatic reactions employing dextransucrase were conducted using sucrose as the donor and various polyols as acceptors, each separately. An optimization process was undertaken through multivariate analysis (Software Design Expert 10.1, StatEase), employing a Central Composite Design (CCD) to investigate optimal conditions within specific ranges for three variables (discrete values of the variables were selected based on preliminary experiments): acceptor-to-donor concentration (1:1; 20–50%, w/v),^36^ reaction time (2 to 48 h), and enzyme activity (0.2–3 U/mL). The aim was to maximize the transglycosylation production (mg/mL) of polyols (refer to Table 1).^37^ Likewise, the yield values (g of polyols-based gluco-oligosaccharides/100 g polyol added) represent the mass of transglucosylated polyol obtained during the synthesis per unit mass of initial polyol. This CCD encompassed 28 initial experiments. Ideally, the catalytic process involves configuring different conditions based on existing literature, including the optimal pH and enzymatic temperature. The predetermined reaction conditions were maintained at 30 °C, utilizing a 20 mM sodium acetate buffer with 0.34 mM CaCl_2_ at pH 5.2.^38^

**Table 1:** Design of Experiments (DOE) Presenting the Experimental Region for the Variables Donor/Acceptor [1:1] (%; w/v), Time of Reaction (Hours) and Enzyme Activity (U/mL) Carried Out for Each Polyol: Xylitol, Erythritol, Maltitol, and Isomaltitol

run	sucrose/polyol [1:1] (%; w/v)	time (h)	activity (U/mL)
1	27.5	13.5	0.9
2	35	25	1.6
3	35	48	1.6
4	50	25	1.6
5	35	25	1.6
6	27.5	13.5	0.9
7	42.5	36.5	0.9
8	35	25	0.2
9	42.5	36.5	2.3
10	27.5	36.5	0.9
11	42.5	13.5	0.9
12	42.5	13.5	0.9
13	35	25	1.6
14	27.5	13.5	2.3
15	35	25	3
16	35	2	1.6
17	35	25	1.6
18	42.5	36.5	2.3
19	42.5	13.5	2.3
20	27.5	36.5	2.3
21	20	25	1.6
22	42.5	13.5	2.3
23	27.5	36.5	2.3
24	35	25	1.6
25	27.5	36.5	0.9
26	35	25	1.6
27	27.5	13.5	2.3
28	42.5	36.5	0.9

To evaluate the relationship between the independent variables ([acceptor/donor]; reaction time; enzyme activity) and the response variable (amount of transglycosylation products), a response surface methodology (RSM) was employed for each polyol. Ultimately, the predicted conditions were experimentally validated in triplicate. The experimental data were analyzed using Design-Expert 11.0 software (Stat-Ease Inc., Minneapolis).^39^

### 2.3 Quantitation by Gas Chromatography coupled to Flame Ionization Detector (GC-FID)

The concentration of transglucosylated products during the design optimization was determined as trimethylsilylated oximes (TMSOs) using Gas Chromatography (GC) with flame ionization detection (FID) as the detection system following the methodology of Gallego-Lobillo et al.^40^ The analysis was carried out on an Agilent Technologies 7820A gas chromatography system with a capillary column DC-5HT (5% phenyl methylpolysiloxane, 30 m × 0.25 mm × 0.1 μm; Agilent J&W Scientific, Folsom, CA). The injection method utilized split mode (20:1), and nitrogen was employed as the carrier gas with a flow rate of 1 mL/min. The initial oven temperature was set at 150 °C and gradually ramped at a rate of 3 °C/min until reaching a final temperature of 380 °C. The total analysis duration was 76.7 min. The FID detector and injection port temperatures were set at 385 and 280 °C, respectively.^40^

The calibration curve was established by injecting carbohydrate standards (glucose, maltose, maltotriose, maltotetraose, and maltopentaose; 0.01–0.5 mg/mL) into the GC-FID system, with peak areas measured to determine the respective response factors. Phenyl-β-d-glucopyranoside (0.5 mg/mL) was employed as an internal standard for quantifying the samples. The calibration standard samples were equally derivatized by TMSOs prior to GC injection.

### 2.4 Matrix-assisted Laser Desorption/Ionization Time-of-flight Mass Spectrometry (MALDI-TOF MS)

The matrix-assisted laser desorption ionization time-of-flight (MALDI-TOF) spectra were recorded using a Bruker Ultraflex II MALDI-TOF instrument (BrukerDaltonics, Bremen, Germany) operating in the linear positive ion mode. Mass spectra ([M + Na]^+^) were obtained over the *m*/*z* range of 300–3000. For each sample, 1 μL was mixed with 0.4 μL of 1 mM NaCl and 1 μL of 2,5-Dihydroxybenzoic acid (20 mg/mL in 70% ACN and 0.1% TFA). Subsequently, 0.5 μL of the mixture was applied to a stainless-steel sample plate (Applied Biosystems, Foster City, CA) and dried under vacuum. The resulting mass spectrum was generated by averaging 100 laser shots per spot. Malto-oligosaccharides were utilized for instrument calibration.

### 2.5 Purification of Transglucosylated Polyols by Size Exclusion Chromatography (SEC)

SEC was utilized to purify the samples obtained under the optimal synthesis conditions as per the DOE, following the methodology by Hernandez et al. Briefly, the Bio-Gel P2 (Bio-Rad, Hercules, CA) stationary phase was packed in a glass column (90 cm × 1.6 cm) and equilibrated with 0.02% sodium azide (mobile phase). Two milliliters of each sample were run at a flow rate of 0.25 mL/min, and after the elution of the void volume (170 mL), 80 samples of 5 mL each were collected. All fractions underwent analysis using direct infusion ESI-MS with a Triple-Quadrupole Agilent 6500 QQQ (Folsom, CA) in positive mode. The mobile phase was composed of 50% v/v acetonitrile in 0.1% v/v formic acid at a flow rate of 0.3 mL/min. The sheath gas was set to 12 L/min at 300 °C, the drying gas to 8 L/min at 220 °C, the nebulizer pressure to 40 psi, and the capillary voltage to 3000 V. Fractions exhibiting a single degree of polymerization with the highest purity, characterized by the absence of other transglucosylated polyol ions, underwent NMR analysis.^41^

### 2.6 Nuclear Magnetic Resonance (NMR)

NMR spectra were recorded at 298 K, using D_2_O as solvent, on an Agilent SYSTEM 500 NMR spectrometer (^1^H 500 MHz, ^13^C 125 MHz) equipped with a 5 mm HCN cold probe. Chemical shifts of ^1^H (δH) and ^13^C (δC) in parts per million were determined relative to internal standards of sodium [2,2,3,3-^2^H_4_]-3-(trimethylsilyl)-propanoate in D_2_O (δH 0.00) and 1,4-dioxane (δC 67.40) in D_2_O, respectively. One-dimensional (1D) NMR experiments (^1^H and ^13^C{^1^H}) were performed using standard pulse sequences. Two-dimensional (2D) [^1^H, ^1^H] NMR experiments [gradient correlation spectroscopy (gCOSY) and total correlation spectroscopy (TOCSY)] were carried out with the following parameters: delay time of 1 s, spectral width of 2800 Hz in both dimensions, 2048 complex points in t2, 4 transients for each of 128 (200 for TOCSY) time increments, and linear prediction to 512. The data were zero-filled to 2048 × 2048 real points. 2D [^1^H–^13^C] NMR experiments [gradient heteronuclear single-quantum coherence (gHSQC)], hybrid experiment gHSQC-TOCSY used the same ^1^H spectral window, a ^13^C spectral window of 7541.5 Hz, 1 s of relaxation delay, 1024 data points, and 128- or 200-time increments, with a linear prediction to 256. The data were zero-filled to 2048 × 2048 real points. Typical numbers of transients per increment were 4 and 16. A mixing time of 80 ms was used for gHSQC-TOCSY experiment.

## 3 Results

### 3.1 Optimization of Enzymatic Synthesis Conditions by CCD

GC-FID was employed in the screening process to monitor the progress of the enzymatic glucosylation of the tested polyols. The CCD was conducted to assess the impact of the three independent variables (% donor:acceptor, reaction time, and enzyme activity) on the yield and concentration of transglucosylated polyols (mg/mL) as the response variable. Table S1 illustrates the concentrations of new peaks obtained for each polyol, calculated from GC-FID analyses. The CCD encompassed 28 runs for each polyol, with 2 replicates of factorial points and 6 at the central point. An optimization phase was executed through the application of response surface methodology (RSM) to enhance product formation.

Analysis of variance (ANOVA) was performed to determine the significance and adequacy of the regression model fit. The statistical significance of the model was established at *p* ≤ 0.05. For each fitted model (linear with interactions for xylitol, erythritol and maltitol; and quadratic for isomaltitol), *p* < 0.0001, and for the lack higher than 0.3, indicating the models’ high adequacy and significance. Furthermore, the determination coefficients (*R*^2^) for each model were 0.96, 0.90, 0.85, and 0.82 for new peaks of xylitol, erythritol, maltitol, and isomaltitol, respectively. Additionally, the coefficients of variation (CV %) were under 10%, indicating acceptable and satisfactory variation.

**Figure 1:**
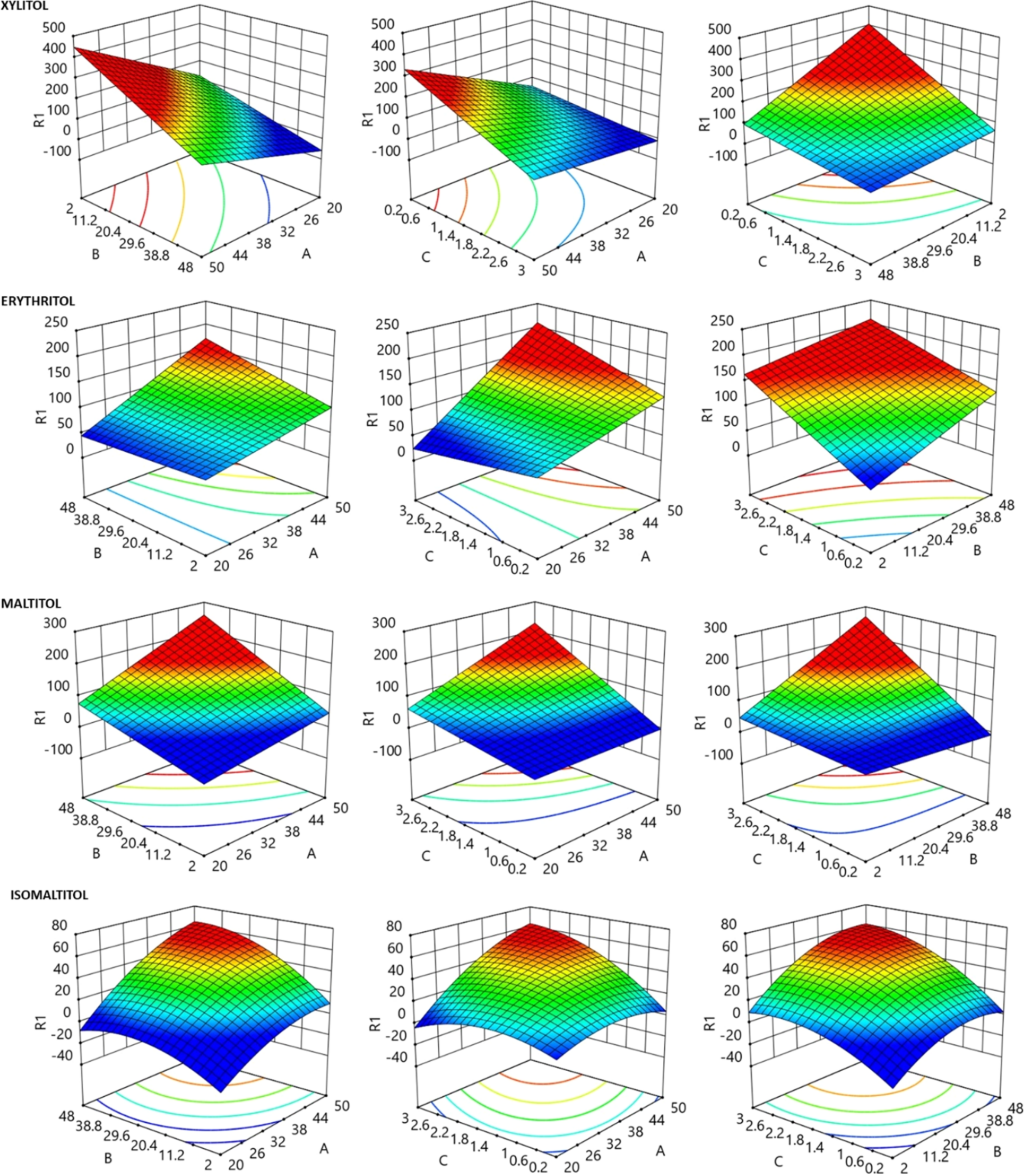
Three-dimensional plots showing the maximization of the yield of new peaks (R1) formation for each polyol. Variables: (factor A; %) sucrose/polyol [1:1] (w/v) concentration, (factor B; hours) time of reaction and (factor C; U/mL) enzyme activity.

Figure 1 presents the response surface obtained for the yield of new peaks for each polyol. The coordinates yielding the maximum were *A*: 45.52%, *B*: 19.15 h, *C*: 0.27 U/mL for erythritol; *A*: 49.91%, *B*: 47.41 h, *C*: 1.84 U/mL for maltitol; *A*: 49.96%, *B*: 40.05 h, *C*: 2.90 U/mL for isomaltitol; and *A*: 47.43%, *B*: 38.92 h, *C*: 2.60 U/mL for xylitol. The relationship between the response evaluated and the variables for each polyol was fitted into the following polynomial equations:
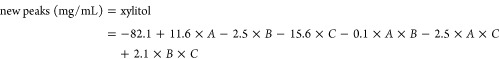

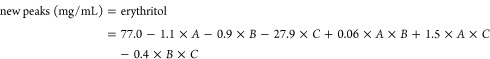

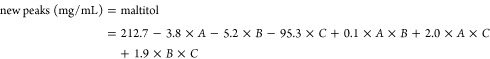

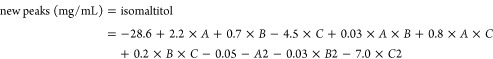
The individual response values and their respective confidence intervals are depicted in Table 2. To validate these predictive models, optimal conditions were experimentally assessed through three replicates, and these showed no significant differences from the theoretical results (Table 2). Figures S17–S20 show specific GC-FID chromatograms for each polyol studied in the optimal conditions obtained from the DOE.

**Table 2:** Criteria for the Optimization Obtained from the Model Equations for Each Polyol in Order to Maximize the Experimental Response

			confidence interval^d^		
response	theoretical result^a^^e^	experimental result^b^,^c^	(−)	(+)	yield (%)^e^
glucosylated xylitol (mg/mL)	286.5	282.4 ± 4.8	262.3	310.7	59.5 ± 1.0
glucosylated erythritol (mg/mL)	169.6	171.9 ± 1.7	146.6	192.7	37.8 ± 0.4
glucosylated maltitol (mg/mL)	220.6	217.4 ± 2.3	174.9	266.2	43.6 ± 0.5
glucosylated isomaltitol (mg/mL)	60.1	61.9 ± 2.6	41.7	78.8	12.4 ± 0.5

a Obtained from model prediction at the optimal settings.

b Obtained from an average of additional three runs conducted at the optimal settings.

c Standard deviations and relative standard deviations (*n* = 3) of experimental results are also represented.

d Lower (−) and upper (+) confidence interval values calculated to a confidence level of 95%.

e Refers to mg glycosylated polyol/100 mg of initial polyol.

### 3.2 Structural Characterization by MALDI-TOF

**Figure 2:**
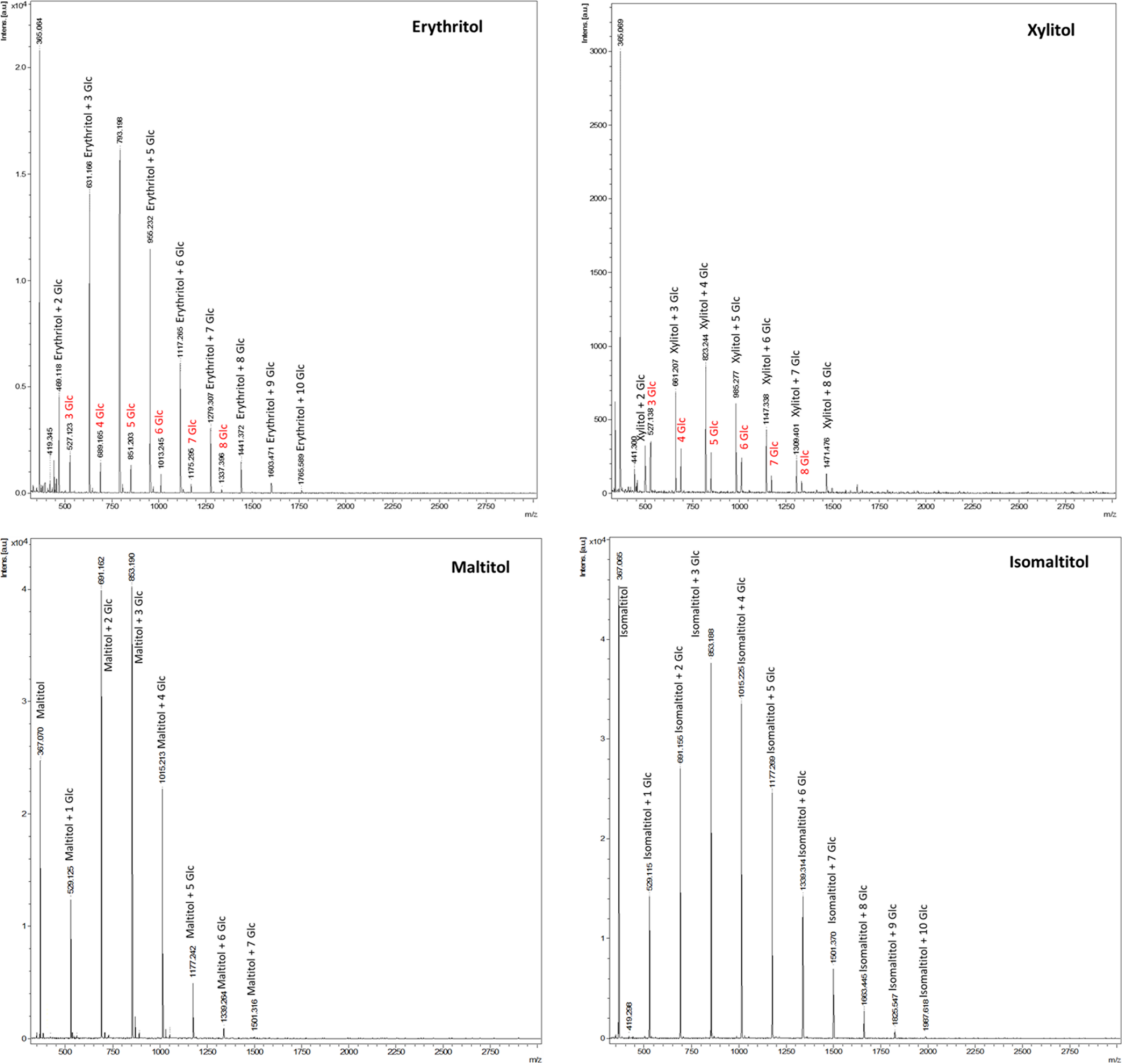
MALDI-TOF profiles of the new transglucosylated polyols.

Taking into consideration the *m*/*z* values obtained from MALDI-TOF, higher glucosylated chains than the ones characterized by NMR and/or detected by GC-FID were observed for each polyol. Figure 2 illustrates the spectra of newly glucosylated erythritol, xylitol, maltitol, and isomaltitol, respectively. These glycosylations were evident within the *m*/*z* range of 500–2000. These mass values indicate the attachment of up to 10 glucose units to erythritol, 8 glucose units to xylitol, 7 glucose units to maltitol, and 10 glucose units to isomaltitol, as shown in the profiles depicted in Figure 2. Interestingly, gluco-oligosaccharides without any polyol attachment (*m*/*z* 527, 689, 851, 1013, 1175, and 1337–from DP3 to DP8) were found in the samples containing erythritol and xylitol as acceptors and not in the samples containing maltitol and isomaltitol. These results could indicate that the enzyme’s affinity to transfer glucose units is higher for the two monomeric polyols (maltitol and isomaltitol) than for sucrose when used as an acceptor.

Given that the structure of an individual DP was characterized by NMR, and considering the reported transglucosylation activity of dextransucrase, as well as the observed *m*/*z* values by MALDI-TOF, it is plausible that the other DPs found consist of glucose units linked by α(1 → 6) linkages.

### 3.3 Structural Characterization by NMR

In order to perform reliable NMR identification and due to the complexity of the carbohydrate mixture in the transglucosylated polyol samples, a prior purification by SEC was carried out. After SEC, 80 fractions per sample were obtained and analyzed by MS using direct infusion in a triple-quadrupole. The chosen samples were those with the highest abundance of ions for a single transglucosylated polyol and low or absent levels of other transglucosylated polyols. In the case of isomaltitol, maltitol, and xylitol, the most pure and abundant reaction was the corresponding to the polyol transglucosylated with 4 units of glucose (Figures S21, S22, and S23). For erythritol, the most pure and abundant reaction was this polyol transglucosylated with 5 glucose units (Figure S24).

Complete structural elucidation of the fractions from the four studied polyols was carried out by the combined use of 1D and 2D [^1^H–^1^H] and [^1^H–^13^C] NMR experiments (gCOSY, TOCSY, multiplicity-edited gHSQC, gHSQC-TOCSY and gHMBC). ^1^H and ^13^C NMR assignments for identified compounds are given in Table 3. A full set of spectra are collected in the Supporting Information.

**Table 3:**
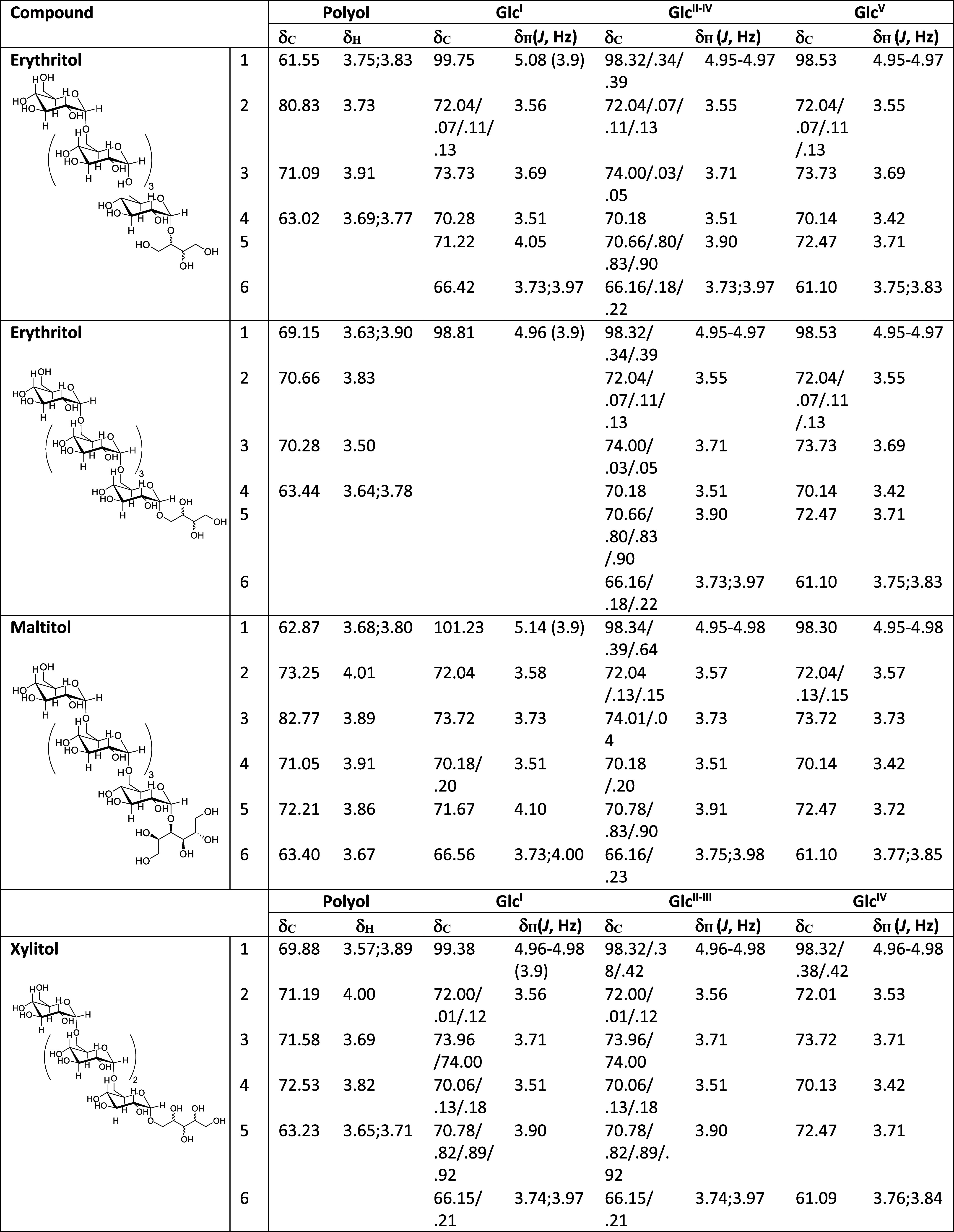

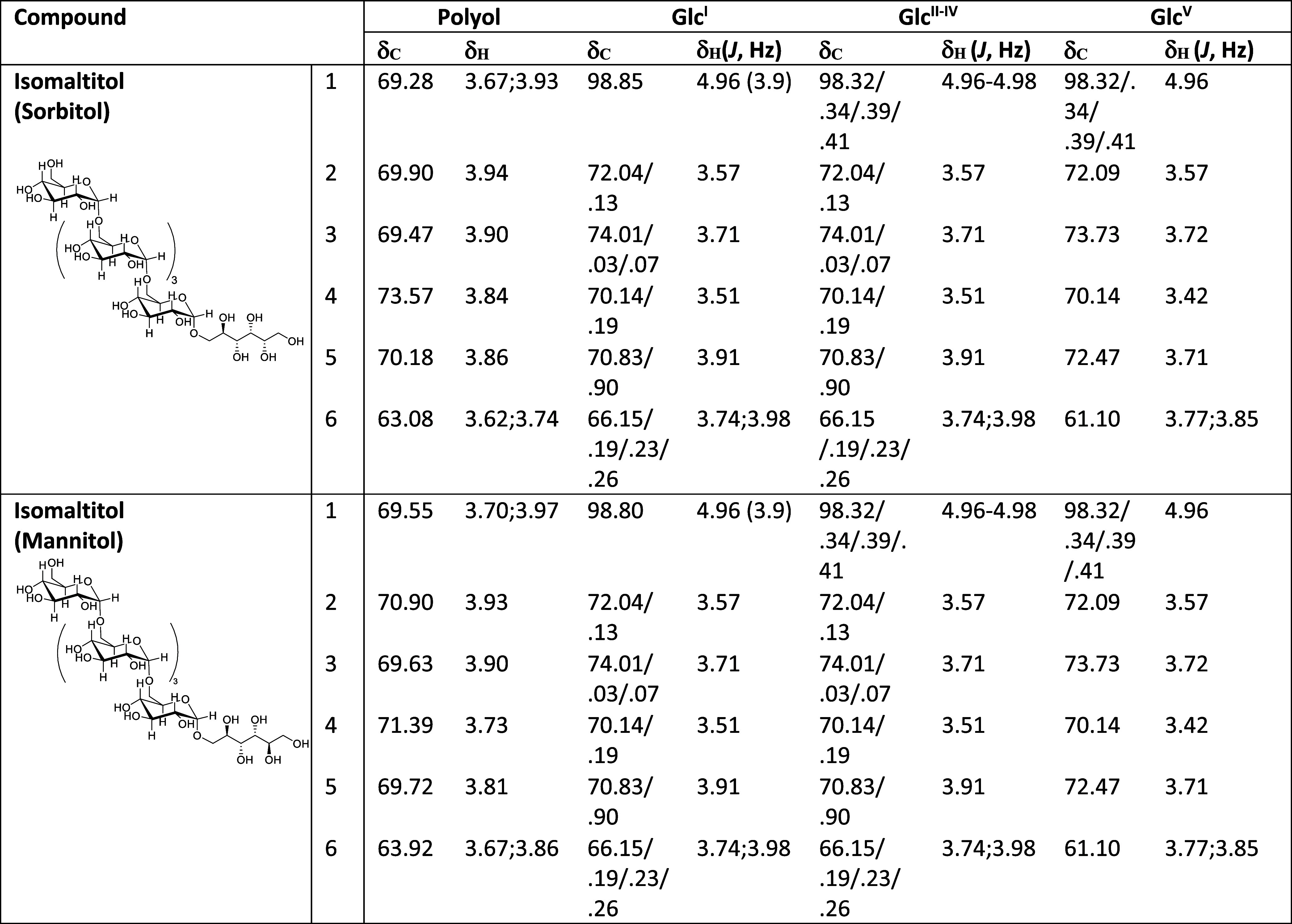
NMR Results of the Optimal Conditions Gotten from the DOE Process for Each Polyol

**Figure 3:**
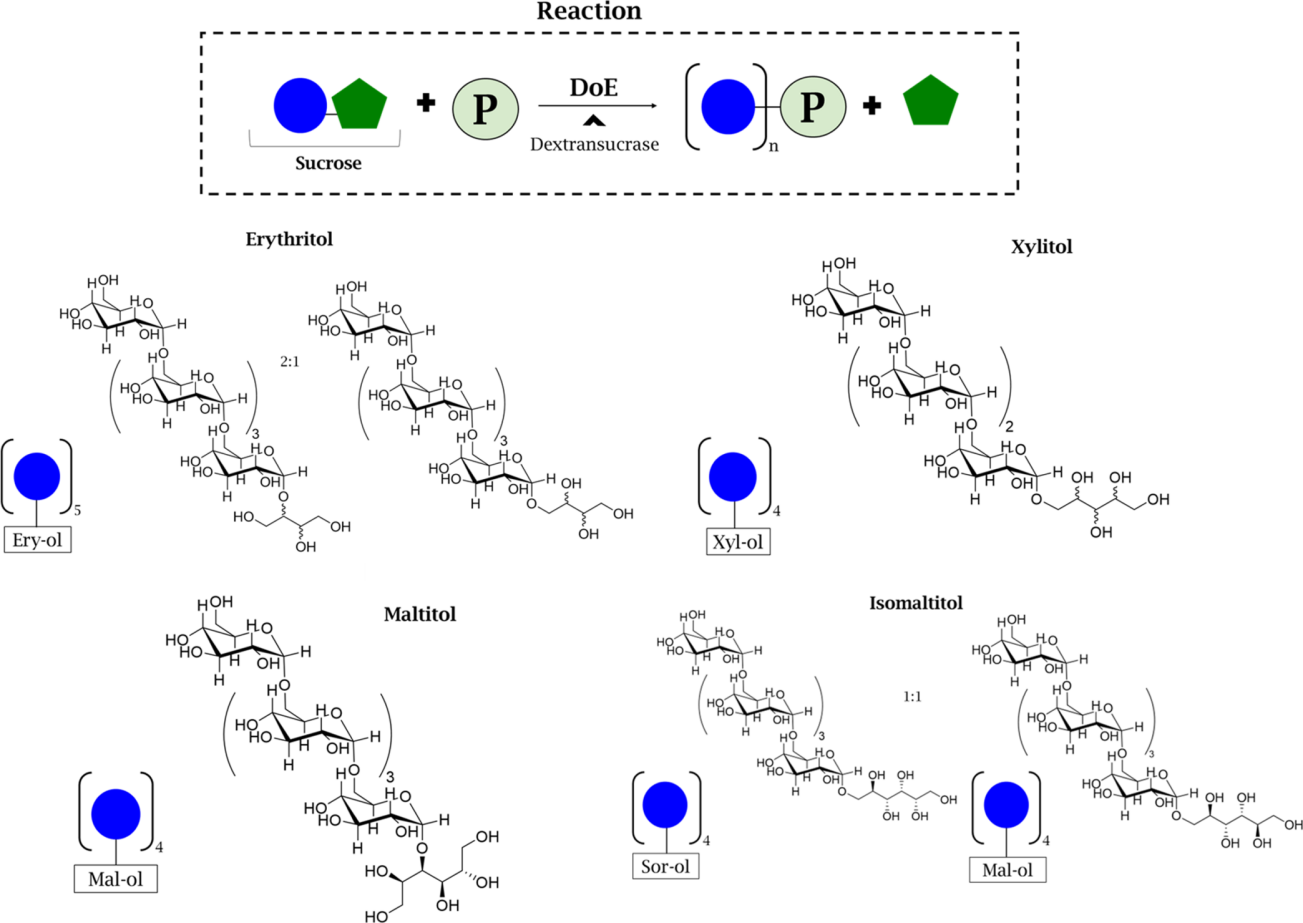
Scheme of catalysis carried out with dextransucrase from *L. mesenteroides* and the structure elucidations by NMR of the polyols after the optimization reaction.

For erythritol, the ^1^H NMR and ^13^C spectra showed the existence of a mixture of two compounds, one where the terminal glucose binds to a primary hydroxyl and the other to a secondary hydroxyl of erythritol, at a 1:2 rate. A multiplicity-edited gHSQC spectrum was used to determine proton-carbon single bond correlations, indicating the presence of a chain of five glucose units for both major and minor compounds, all of them with α configuration [*J* (H1, H2) = 3.9 Hz]. Results from gCOSY, TOCSY, gHSQC-TOCSY and gHMBC revealed the presence of sugar moieties and the existence of two erythritol units. From these results, we also determined the existence of the two anomeric carbons and protons directly linked to both units of erythritol, (99.75, 5.08 ppm for the secondary hydroxyl binding and 98.81, 4.96 ppm for the primary). The linkage between the erythritol unit and the sugar was established from gHMBC correlations between the anomeric proton at δH 5.08 and the erythritol methyne central carbon at δC 80.83 in major compound, and between the anomeric proton at δH 4.96 and the erythritol methylene carbon at δC 69.15 in minor compound (see Figures S1 and S2 from Supporting Information). Also, gHMBC correlations lead us to establish a α(1 → 6) linkage between glucoses. Consequently, the following structures were deduced for both compounds (Figure 3). The stereochemistry of stereogenic centers of erythritol is not defined, due to we are not able to distinguish if the linkage has occurred with the *R* or *S* center in the major isomer or with hydroxyl 1 or 4 in the minor isomer (Figure S4). Each pair would provide the same NMR signals in both cases, so probably we have a mixture of both in each case, in a percentage that cannot be estimated.

Following the same procedure, the ^1^H NMR and ^13^C spectra of the fraction from xylitol showed the existence of a single compound. gCOSY, TOCSY and gHSQC spectra were consistent with the presence of four glucoses and a xylitol moiety. In this case, *J* value of the anomeric protons could not be measured, due to signal overlap, but an α configuration was assigned by comparison of chemical shifts with those from erythritol derivatives. The linkage between xylitol and glucose moieties was established from gHMBC correlations (see Figure S5 from Supporting Information). So, relevant correlation peaks between the glucosyl anomeric proton (δH 4.96–4.98) and the methylene xylitol moiety carbon (δC1 69.88) and between the corresponding methylene protons (δH 3.57) and the glucosyl anomeric carbon (δC 99.38) were observed. Again, gHMBC correlations lead us to establish a α(1 → 6) linkage between glucoses. Taking into account that glycosylation would make the xylitol residue asymmetric, it would thus lead to the formation of a mixture of two diastereomeric glucosyl-xylitol molecules, indistinguishable by NMR (Figure 3).

In the case of maltitol, it is described as 4-*O*-α-d-glucopyranosyl-d-glucitol, a residue whose presence was confirmed by mono- and bidimensional experiments and chemical shifts are in agreement with those described in literature.^42^ The linkage between the glucose ring and the glucitol residue was corroborated by gHMBC correlations (see Figure S9 from Supporting Information) showing a correlation peak between the glucosyl anomeric proton (δH 5.14) and the methylene glucitol moiety carbon (δC1 82.77). In^13^C spectrum, signals corresponding to 5 anomeric carbons can be observed (δC1 98.30, 98.34, 98.39, 98.64 and 101.23). Those, together with gHMBC correlations between anomeric protons and carbons in the area of 66 ppm (see Figure S11 from Supporting Information), confirmed the linkage between a chain of four glucoses with a α(1 → 6) linkage and the maltitol moiety (Figure 3). In this case, the stereochemistry of the polyol residue is prefixed by the stereochemistry of maltitol.

Last, isomaltitol is described as an equimolecular mixture of glucosorbitol and glucomannitol (Figure 3). The presence of these residues was confirmed by mono- and bidimensional experiments and chemical shifts are in agreement with those described in the literature, although according to bidimensional correlations, we have changed the assignments of some positions (see Table 3).^43^

In this case, anomeric carbons belonging to the glucose chain are in the region between 98.32 and 98.41, and two of them which are more deshielding (δC1 98.80 and 98.85) correspond to the two terminal glucoses, which bind to mannitol and sorbitol, respectively. These linkages were corroborated by gHMBC correlations (see Figure S13 from Supporting Information) showing a correlation peak between the glucosyl anomeric proton (δH 4.96 in both cases) and the methylene sorbitol and mannitol moiety carbon (δC1 69.28 and 69.55, respectively), and between the methylene protons (δH 3.67, 3.93 for sorbitol and 3.70, 3.97 for mannitol) and the anomeric carbons (δC1 98.85 and 98.80, respectively).

Those, together with gHMBC correlations between anomeric protons and carbons in the area of 66 ppm (see Figure S13 from Supporting Information), confirmed the linkage between a chain of four glucoses with an α(1 → 6) linkage and the glucosorbitol and glucomannitol moieties in a 1:1 rate (Figure 3). In this case, stereochemistry of the polyol residues is also prefixed by the stereochemistry of each unit of isomaltitol.

## 4 Discussion

In the food industry, polyols have established themselves as valuable components due to their technofunctional advantages and providing chemical and microbiological stability, among other attributes.^3^ Regarding their role as sweeteners in the market, they are positioned as substitutes for traditional sugars, presenting a similar sweet flavor profile.^44^ Given their increasing prominence, our study focuses on the enzymatic modification of the most commonly used commercial polyols with the aim of producing a novel sweetener with potential prebiotic properties. Dextransucrases catalyze the synthesis of dextran using sucrose as the donor substrate. In the presence of suitable acceptor molecules like maltose, isomaltose or isomaltulose, they facilitate the synthesis of IMOs, resulting in oligosaccharides primarily linked by α(1 → 6) glycosidic bonds.^45,46^ Moreover, in addition to the evidence regarding the prebiotic properties of IMOs, other beneficial physiological functions such as enhanced bowel function have been investigated by *in vivo* studies.^47^

Consequently, this study focuses on synthesizing prebiotic oligosaccharides structures using dextransucrases, employing an experimental design (DOE) to optimize a high glucosylation yield of polyols.^36^ GC-FID quantitative data (Table S1) reveals new structures with higher degrees of polymerization in the reactions between sucrose as the donor and polyol as the acceptor (Figures S17–S20). Structural characterisations were addressed by incorporating NMR and MALDI-TOF analyses to characterize reactions from the optimal conditions established by the entire experimental design.

NMR confirms the presence of glucose residues linked to polyol via *O*-glycosidic bonds between the hemiacetal group of one glucose unit (C1) and the hydroxyl group (C6-OH) of another glucose unit, forming consecutive α(1 → 6) configuration linkages. The scheme presented in Figure 3 illustrates the actions of dextransucrase according to the NMR findings. MALDI-TOF offers precise mass determinations, revealing larger monomer chains attached to polyol structures, with up to 10 glucose units for erythritol and isomaltitol, 6 glucose units for xylitol, and 7 for maltitol (Figure 2). Due to their unique glucose interunit linkage, these newly synthesized structures can be categorized as polyol-based α-gluco-oligosaccharides.^21^ Prior research has employed enzymatic reactions to produce α(1 → 6) GlcOS, such as IMOs, gentio-oligosaccharides, and cello-oligosaccharides, utilizing dextransucrases from *L. mesenteroides* with various acceptor carbohydrates.^48,49^ Additionally, for reduced reliance on monosaccharides, the synthesis of GlcOS by dextransucrase using steviol glycosides as an acceptor substrate has been explored, as demonstrated by Ko et al.^50^ More recently, Muñoz-Labrador et al. adopted a similar approach using cyclodextrin glucosyltransferases (CGTases), a more commonly employed enzyme in industrial processes.^24^ The use of glucansucrases can be limited by several factors such as enzyme selectivity and efficiency.^22^ For this reason, the standout feature of this study is the incorporation of a DOE approach to optimize the yield response, addressing the principal challenge of enzymatic syntheses, as well as the novelty of the structures obtained and the highly efficient transglucosylation reaction that yielded polyols-based gluco-oligosaccharides of up to 10 glucose units. In addition to the prebiotic effect, the glycosylation of these polyols could influence sensorial characteristics of the glycosylated products since Ruiz-aceituno et al. observed a decrease of the sweetness by the increasing length of the oligosaccharides chain.^51^

Leveraging dextransucrase from *L. mesenteroides* B512F, this study follows a bottom-up method, catalyzing the formation of new glycosidic bonds onto polyols via glycosylation. The glucosyl-glucose α(1 → 6) linkages are, at least, partially resistant to hydrolysis by intestinal enzymes, endowing these newly synthesized compounds with recognized prebiotic properties.^52,53^ The synthesis of prebiotic structures using a sweetener as an acceptor molecule holds the potential for health benefits, akin to the recent work by Muñoz-Labrador et al., which demonstrated significant growth of beneficial bacteria like *Bifidobacterium* through *in vitro* fermentation studies, among other bacterial groups and additional metabolites produced.^23^ Furthermore, as supported by existing literature, polyols play a digestive role similar to prebiotic carbohydrates, especially in those cases where they can be poorly absorbed in the small intestine, reaching the colon intact, stimulating the growth of bacteria or production of metabolites such as acetic, propanoic, and butanoic acids.^10^ However, in the case of low molecular weight polyols, like erythritol or xylitol, the bioavailability to the large intestine microbiome has been undermined, but an efficient transglycosylation process as the one described in our work could overcome the absorption at the small intestine level and become fully accessible for interaction with the colon microbiota.

The regioselective glycosylation of polyols introduces an innovative catalytic approach to producing polyol-sweetening derivatives. Previous work by Zhang et al., explored the glycosylation of a polyol using protein-expressed glucanotransferases.^35^ However, the reaction efficiency yielded monoglucosylation of erythritol. Until now, no prior studies have combined polyols with dextransucrase to explore this novel enzymatic avenue.

In summary, the food industry remains steadfastly focused on the pursuit of novel products that enhance organoleptic properties in food formulations, foster healthier compositions with biofunctional attributes, align with eco-friendly alternatives, and optimize industrial processing wherever feasible. In this context, we have presented a pioneering enzyme development that yields novel variants of glucosylated polyols, utilizing dextransucrase from *L. mesenteroides* and sucrose as the donor substrate. The elucidated structures (polyols with α(1 → 6) glucosyl-glucose units) could potentially serve as new prebiotic sweeteners; however, further organoleptic and biological studies are required to establish their added benefits.

## Supporting Information Available

Quantitative values obtained during the DOE quantified by GC-FID analyses, for each polyol (Table S1); ^1^H NMR (500 MHz, D_2_O) of Erythritol-5Glc (Figure S1); ^13^C NMR (125 MHz, D_2_O) of Erythritol-5Glc (Figure S2); gCOSY and TOCSY (500 MHz, D_2_O) of Erythritol-5Glc (Figure S3); multiplicity-edited gHSQC, g-HSQC-TOCSY and gHMBC (500 MHz, D_2_O) of Erythritol-5Glc (Figure S4); ^1^H NMR (500 MHz, D_2_O) of Xylitol-Glc (Figure S5); ^13^C NMR (125 MHz, D_2_O) of Xylitol-Glc (Figure S6); gCOSY and TOCSY (500 MHz, D_2_O) of Xylitol-Glc (Figure S7); multiplicity-edited gHSQC, g-HSQC-TOCSY and gHMBC (500 MHz, D_2_O) of Xylitol-Glc (Figure S8); ^1^H NMR (500 MHz, D_2_O) of Maltitol-4Glc (Figure S9); ^13^C NMR (125 MHz, D_2_O) of Maltitol-4Glc (Figure S10); gCOSY and TOCSY (500 MHz, D_2_O) of Maltitol-4Glc (Figure S11); multiplicity-edited gHSQC, g-HSQC-TOCSY and gHMBC (500 MHz, D_2_O) of Maltitol-4Glc (Figure S12); ^1^H NMR (500 MHz, D_2_O) of Isomaltitol-4Glc (Figure S13); ^13^C NMR (125 MHz, D_2_O) of Isomaltitol-4Glc (Figure S14); gCOSY and TOCSY (500 MHz, D_2_O) of Isomaltitol-4Glc (Figure S15); multiplicity-edited gHSQC, g-HSQC-TOCSY and gHMBC (500 MHz, D_2_O) of Isomaltitol-4Glc (Figure S16); GC-FID profile of the enzymatic reaction with Dextransucrase using sucrose:erythritol as substrates (Figure S17); GC-FID profile of the enzymatic reaction with Dextransucrase using sucrose:xylitol as substrates (Figure S18); GC-FID profile of the enzymatic reaction with Dextransucrase using sucrose:maltitol as substrates (Figure S19); GC-FID profile of the enzymatic reaction with Dextransucrase using sucrose/isomaltitol as substrates (Figure S20); ESI-MS mass spectra of the selected fraction for isomaltitol purified by SEC and analyzed by NMR (Figure S21); ESI-MS mass spectra of the selected fraction for maltitol purified by SEC and analyzed by NMR (Figure S22); ESI-MS mass spectra of the selected fraction for xylitol purified by SEC and analyzed by NMR (Figure S23); ESI-MS mass spectra of the selected fraction for erythritol purified by SEC and analyzed by NMR (Figure S24); flowchart of the summarized methodology carried out for the study of the catalytic approach of dextransucrase with most commonly dietary polyols (Figure S25) (PDF)
