# Supplementary material for: Design Optimization of a Novel Catalytic Approach for Transglucosylated Isomaltooligosaccharides into Dietary Polyols Structures by Leuconostoc mesenteroides Dextransucrase
Source: J Agric Food Chem. 2024 Sep 18;72(39):21690–701. doi: 10.1021/acs.jafc.4c04222 (PMC11457383; doi:10.1021/acs.jafc.4c04222)
Supplement: Supplementary file 1 — jf4c04222_si_001.pdf [file jf4c04222_si_001.pdf]

Supporting Information

Design Optimization of a Novel Catalytic Approach for Transglucosylated Isomaltooligosaccharides into Dietary Polyols Structures by *Leuconostoc mesenteroides* Dextransucrase

Ana Muñoz-Labrador[1*], Elisa G. Doyagüez[3], Silvana Azcarate[4], Cristina Julio-Gonzalez[1], Daniela Barile[2], F. Javier Moreno[1], Oswaldo Hernandez-Hernandez[1,2]

[1] Institute of Food Science Research, CIAL (CSIC-UAM), Nicolás Cabrera 9, 28049 Madrid, Spain

[2] Department of Food Science and Technology, University of California Davis, Davis, CA, 95616, USA

[3] Centro de Química Orgánica "Lora Tamayo" (CSIC), Juan de la Cierva 3, 28006, Madrid, Spain

[4] Consejo Nacional de Investigaciones Científicas y Técnicas (CONICET), Godoy Cruz 2290 CABA (C1425FQB), Argentina

*Corresponding author: ana.munoz@csic.es

Table S1. Quantitative values obtained during the DOE quantified by GC-FID analyses, for each polyol.

	New peaks (mg/mL)																	
Runs	**Xylitol**				**Erythritol**					**Maltitol**				**Isomaltitol**			
	**DP 2**	**DP 3**	**DP 4**	**DP 5**	**DP 2**	**DP 3**	**DP 4**	**DP 5**	**DP 6**	**DP 3**	**DP 4**	**DP 5**	**DP 6**	**DP 3**	**DP 4**	**DP 5**	**DP 6**
**1**	111.9	6.7	9.3	4.1	25.4	8.7	14.3	17.1	8.0	7.1	21.7	14.8	7.9	6.5	6.0	6.2	5.0
**2**	59.4	5.9	8.1	5.7	33.8	9.5	21.1	21.7	9.7	11.8	21.8	15.6	9.2	7.8	11.4	12.4	8.9
**3**	30.5	4.1	12.4	2.8	38.4	12.8	26.5	27.3	0.0	8.1	19.9	19.6	17.1	17.3	10.7	10.9	8.3
**4**	218.1	11.1	21.3	6.0	36.6	11.4	21.8	15.8	5.7	78.4	15.4	34.4	26.2	9.8	11.7	15.4	9.1
**5**	77.1	7.6	13.5	4.7	34.4	13.4	23.3	19.2	7.5	72.1	12.8	27.0	15.3	6.7	9.2	13.9	11.9
**6**	102.4	6.3	5.5	0.0	20.0	5.9	13.0	9.7	6.6	67.9	8.3	21.0	13.9	5.7	8.7	8.8	5.6
**7**	97.5	8.8	14.5	4.4	37.5	10.6	25.8	23.1	15.2	11.1	19.9	7.6	2.6	9.4	8.6	7.3	2.9
**8**	246.0	4.9	7.8	3.7	10.6	3.2	3.5	0.0	0.0	97.3	9.7	11.3	1.9	6.3	5.6	4.3	2.5
**9**	29.3	5.6	13.8	3.4	36.2	11.7	11.7	28.4	39.9	81.8	14.5	37.3	23.9	7.2	12.3	13.3	8.6
**10**	15.1	6.1	11.2	1.9	28.9	8.8	16.8	17.6	8.7	7.6	15.6	9.8	2.1	2.6	5.9	7.9	6.9
**11**	215.0	8.2	12.1	4.3	29.5	9.0	17.2	12.6	5.3	13.3	19.8	4.2	2.6	5.9	8.6	6.2	2.8
**12**	210.2	8.3	12.5	4.0	30.7	9.3	18.3	16.2	5.9	10.6	25.3	10.8	4.0	5.6	6.8	2.8	1.6
**13**	76.6	9.4	12.2	5.0	40.7	9.3	21.4	21.2	11.7	7.3	23.9	16.3	4.1	6.9	8.3	8.6	7.7
**14**	13.6	4.6	7.8	3.8	30.2	5.9	13.7	16.3	9.5	8.1	19.7	16.0	3.9	2.2	8.7	7.2	3.3
**15**	21.8	4.1	11.9	4.1	33.6	11.2	22.8	26.4	11.6	12.6	26.4	20.6	10.6	6.6	11.5	10.1	6.9
**16**	0.0	8.6	4.1	1.9	17.7	5.0	8.5	2.9	0.0	1.1	2.2	1.2	0.7	1.1	0.9	1.1	0.7
**17**	34.8	11.0	13.6	6.3	34.0	9.1	20.6	16.7	6.2	6.2	23.6	13.1	6.5	3.7	10.8	13.5	10.6
**18**	26.9	10.5	14.1	4.2	44.6	16.8	32.1	37.6	24.1	94.3	11.7	14.2	3.6	6.7	16.1	16.7	16.1
**19**	84.4	10.9	15.2	4.2	37.7	14.4	29.3	30.4	21.3	13.2	26.0	19.1	6.5	7.5	13.5	11.2	9.8
**20**	14.7	3.2	4.1	0.0	25.0	7.9	14.0	36.9	6.1	71.8	3.7	7.1	18.5	9.3	6.8	8.2	6.5
**21**	9.4	1.5	2.7	1.8	17.4	4.6	11.8	10.3	3.8	6.0	13.7	9.3	3.1	3.1	2.9	4.5	3.3
**22**	80.5	8.3	13.5	4.8	35.0	8.5	25.7	27.9	17.7	18.2	28.5	17.4	7.1	6.6	7.8	10.0	7.2
**23**	14.1	6.6	7.6	4.0	14.5	9.2	15.1	16.5	8.4	11.1	21.1	17.2	7.4	6.0	8.0	8.4	4.6
**24**	80.5	7.5	12.1	4.4	36.0	10.8	21.4	18.7	9.7	9.5	23.6	18.0	5.9	9.3	9.2	12.5	8.1
**25**	14.2	6.4	10.8	2.0	26.7	7.6	17.4	1.9	11.2	6.2	16.3	7.6	5.5	2.6	5.7	7.1	4.8
**26**	79.2	7.8	12.5	4.5	34.9	4.8	21.8	20.4	7.7	10.7	26.6	19.7	10.4	10.7	12.6	14.7	10.4
**27**	26.4	4.5	9.3	3.7	25.1	7.3	16.2	16.9	10.6	6.3	16.1	12.8	7.4	4.2	6.5	5.7	3.6
**28**	93.5	8.5	15.8	4.5	35.8	13.1	24.4	22.1	13.0	11.8	25.0	9.6	2.5	9.2	9.9	7.3	5.9

NMR spectra:

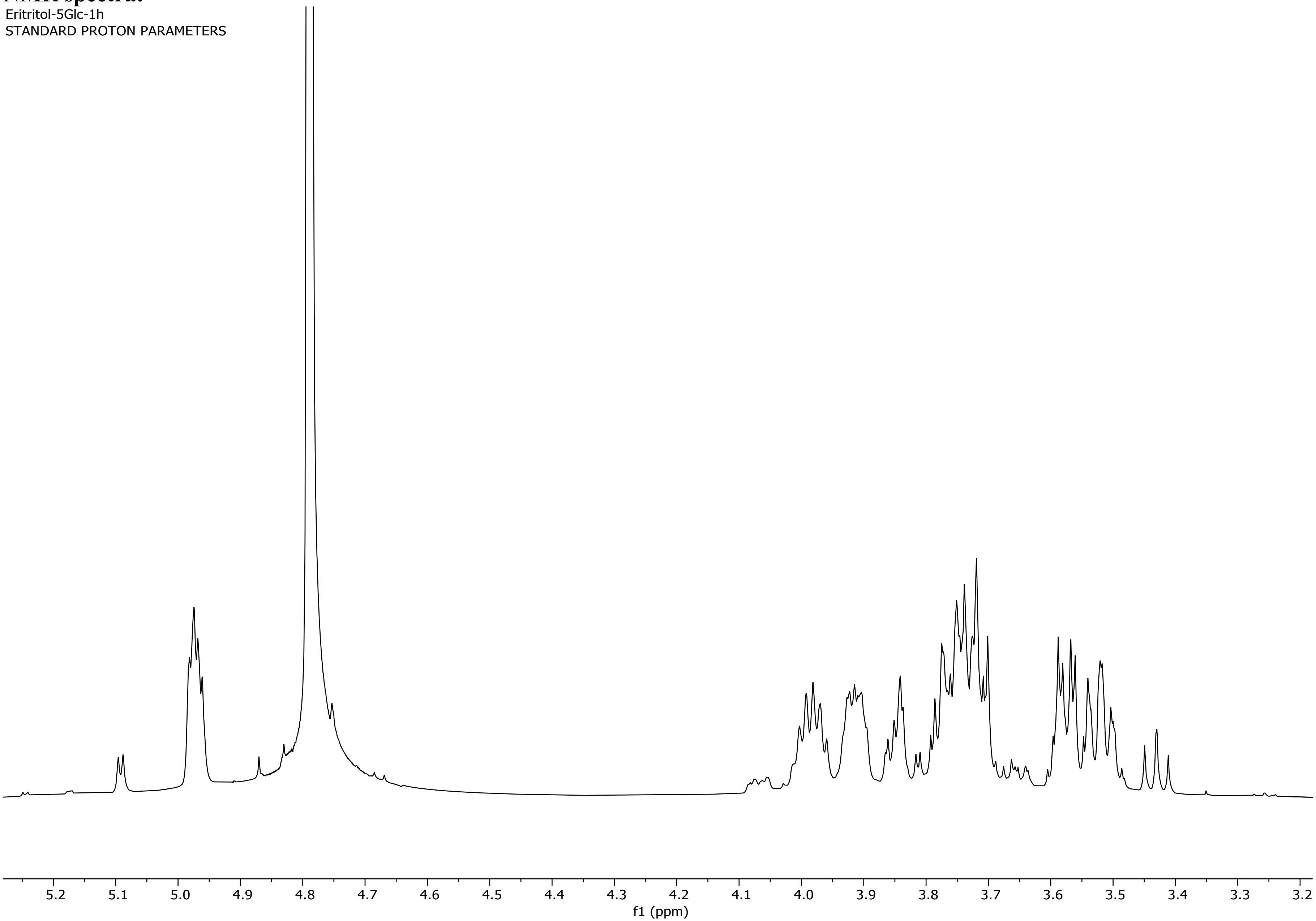

Figure S1. ^{1}H NMR (500 MHz, D_2O) of Erythritol-5Glc.

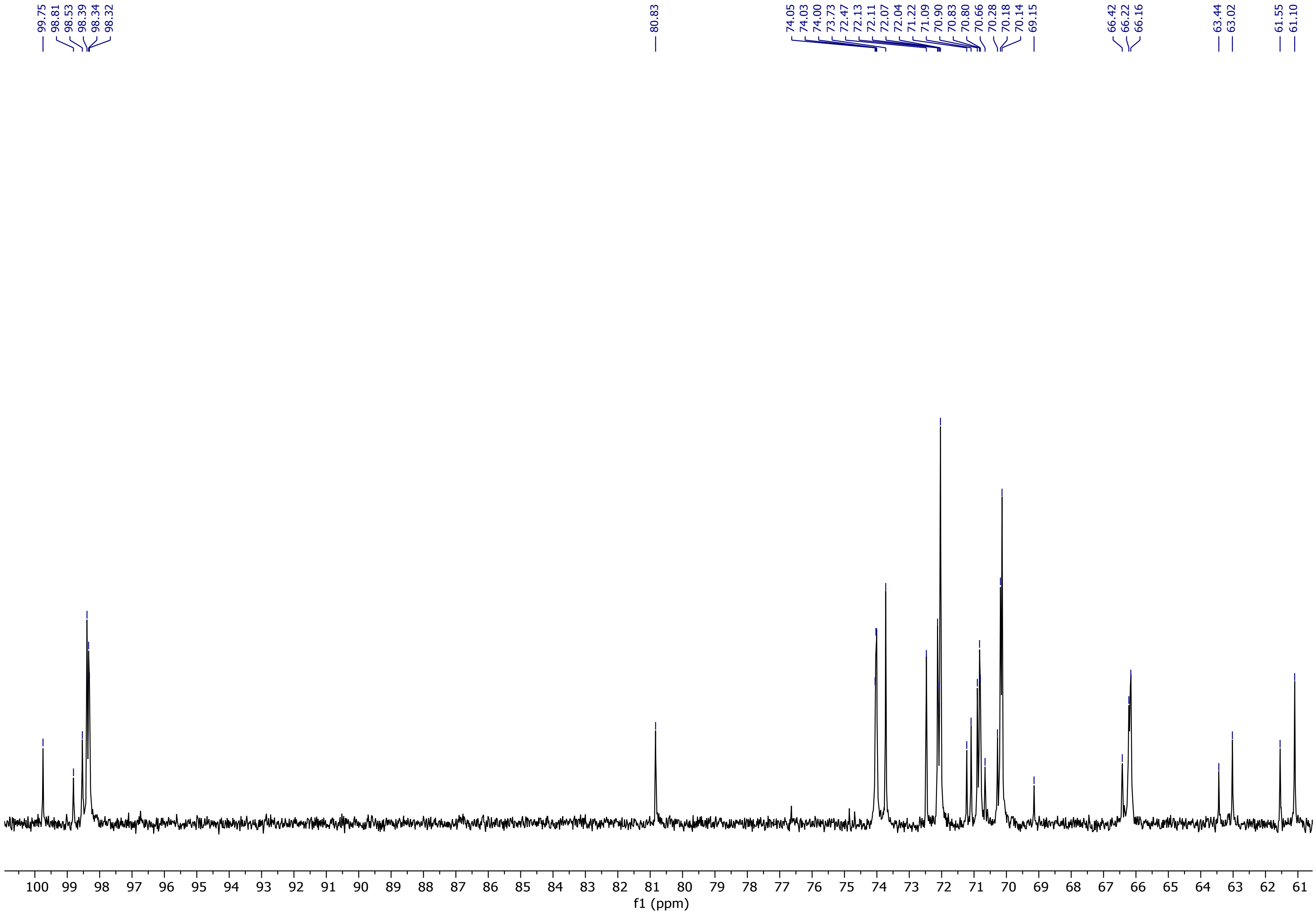

Figure S2. ^{13}C NMR (125 MHz, D_2O) of Erythritol-5Glc.

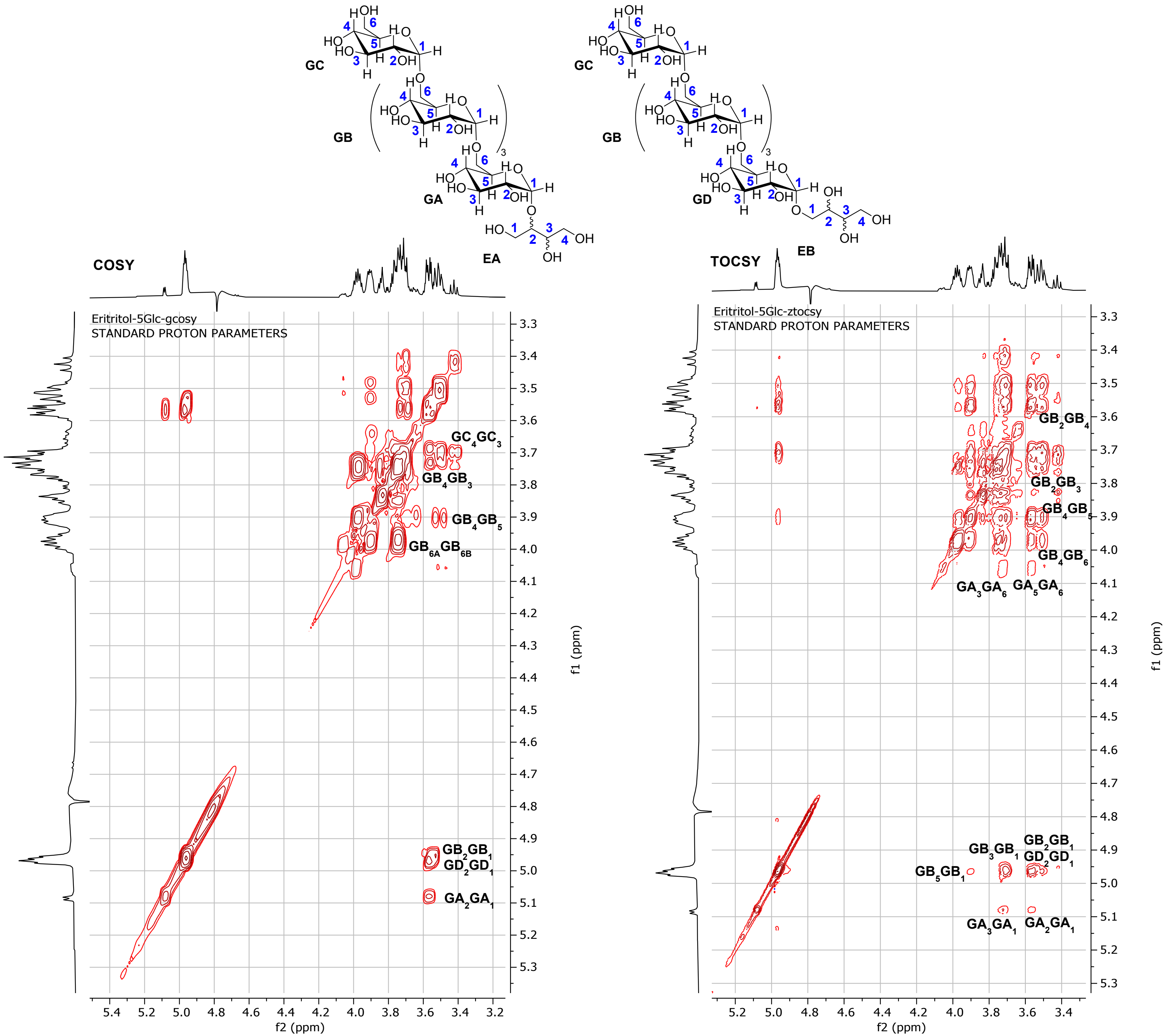

Figure S3. gCOSY and TOCSY (500 MHz, D_2O) of Erythritol-5Glc.

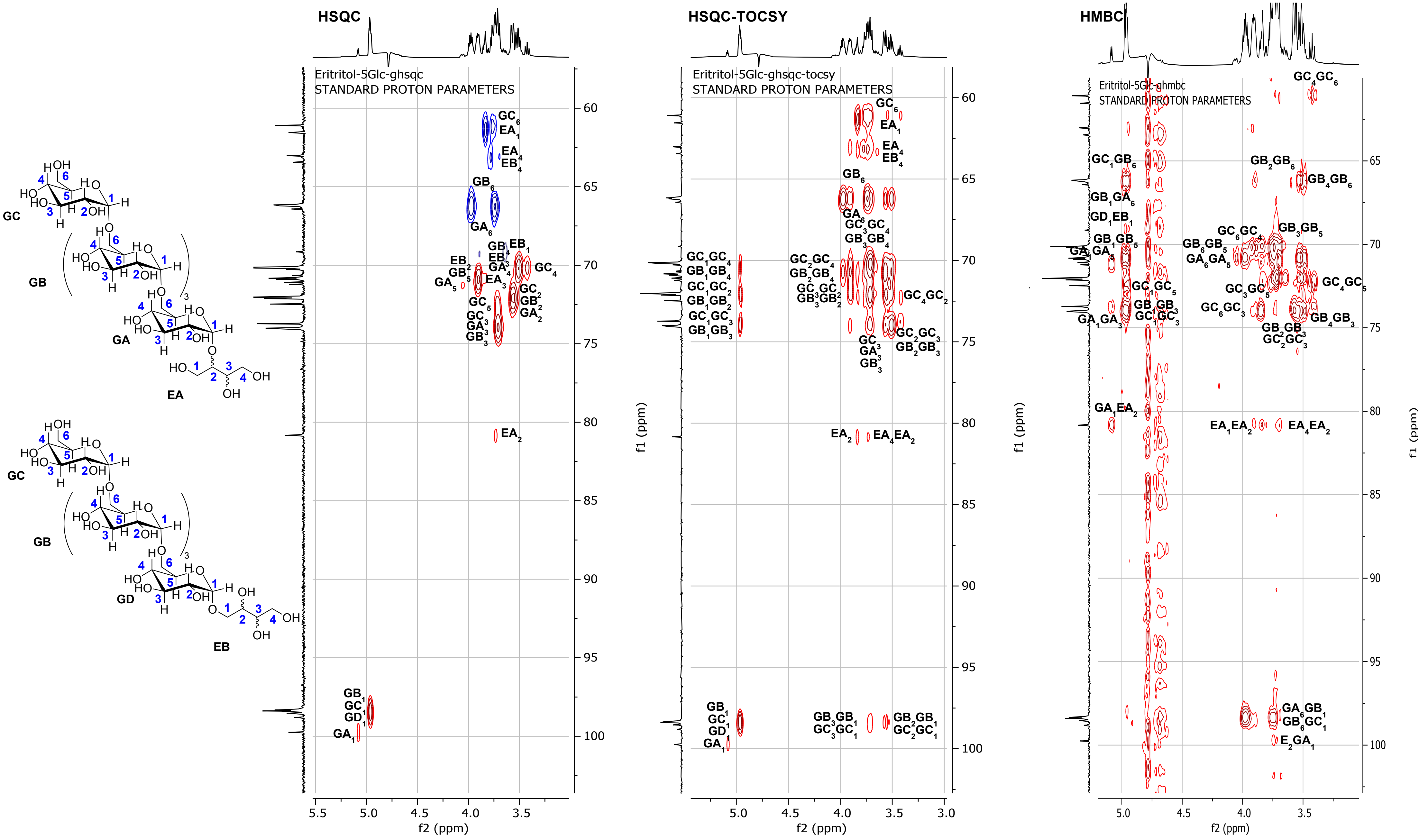

Figure S4. Multiplicity-edited gHSQC (methylene**:** blue cross peaks; methine**:** red cross peaks), gHSQC-TOCSY and gHMBC (500 MHz, D_2O) of Erythritol-5Glc.

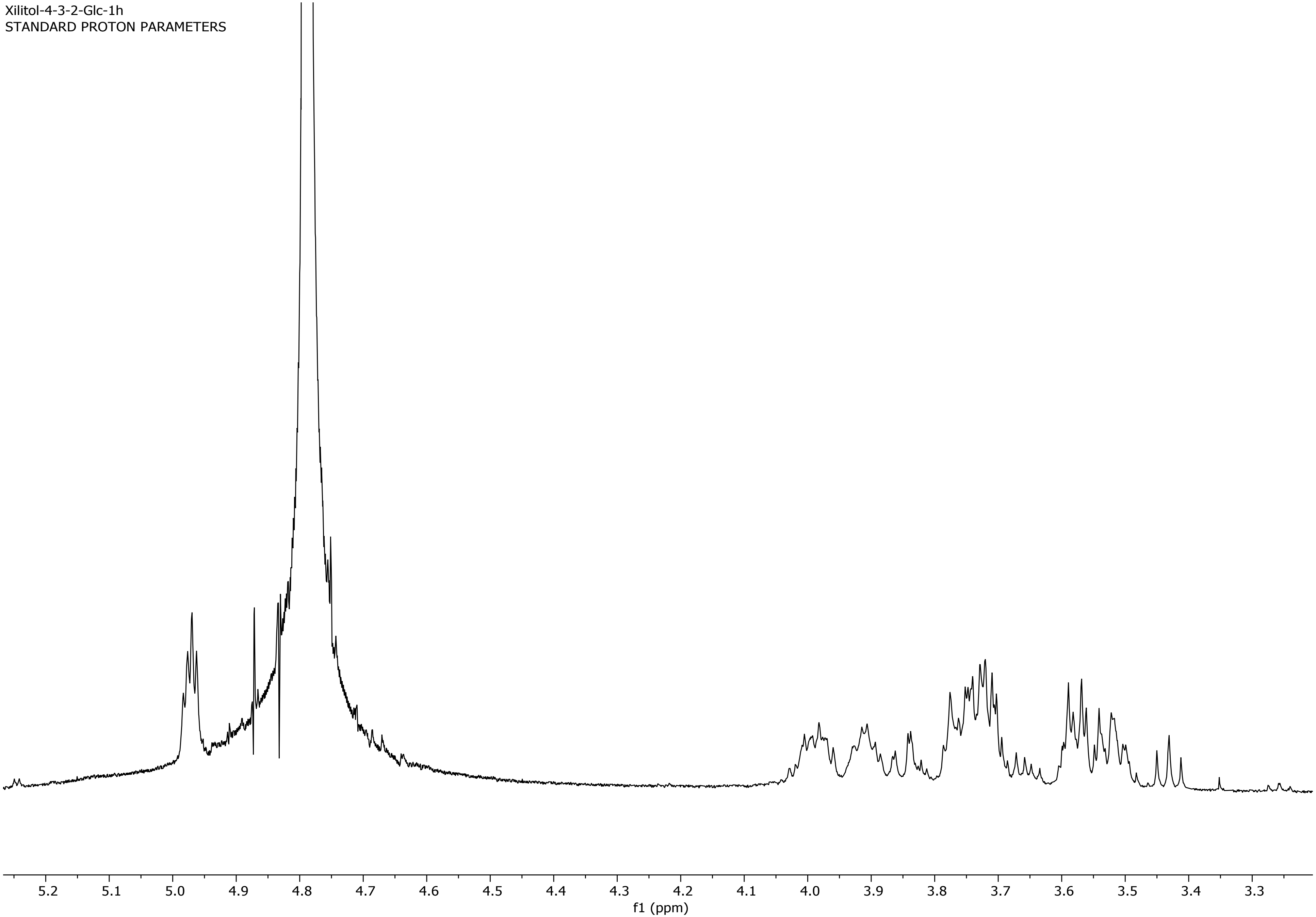

Figure S5. ^{1}H NMR (500 MHz, D_2O) of Xylitol-Glc.

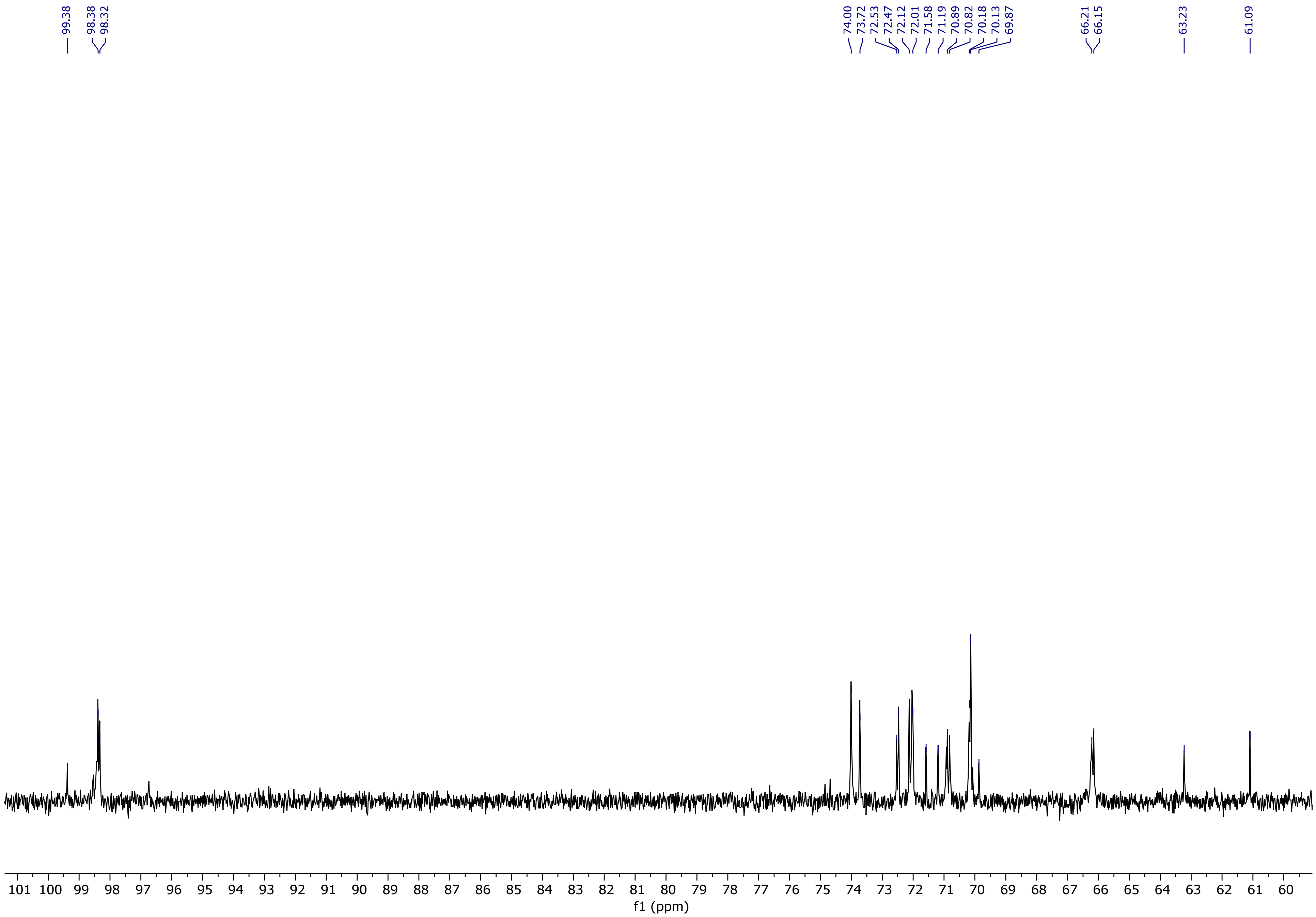

Figure S6. ^{13}C NMR (125 MHz, D_2O) of Xylitol-Glc.

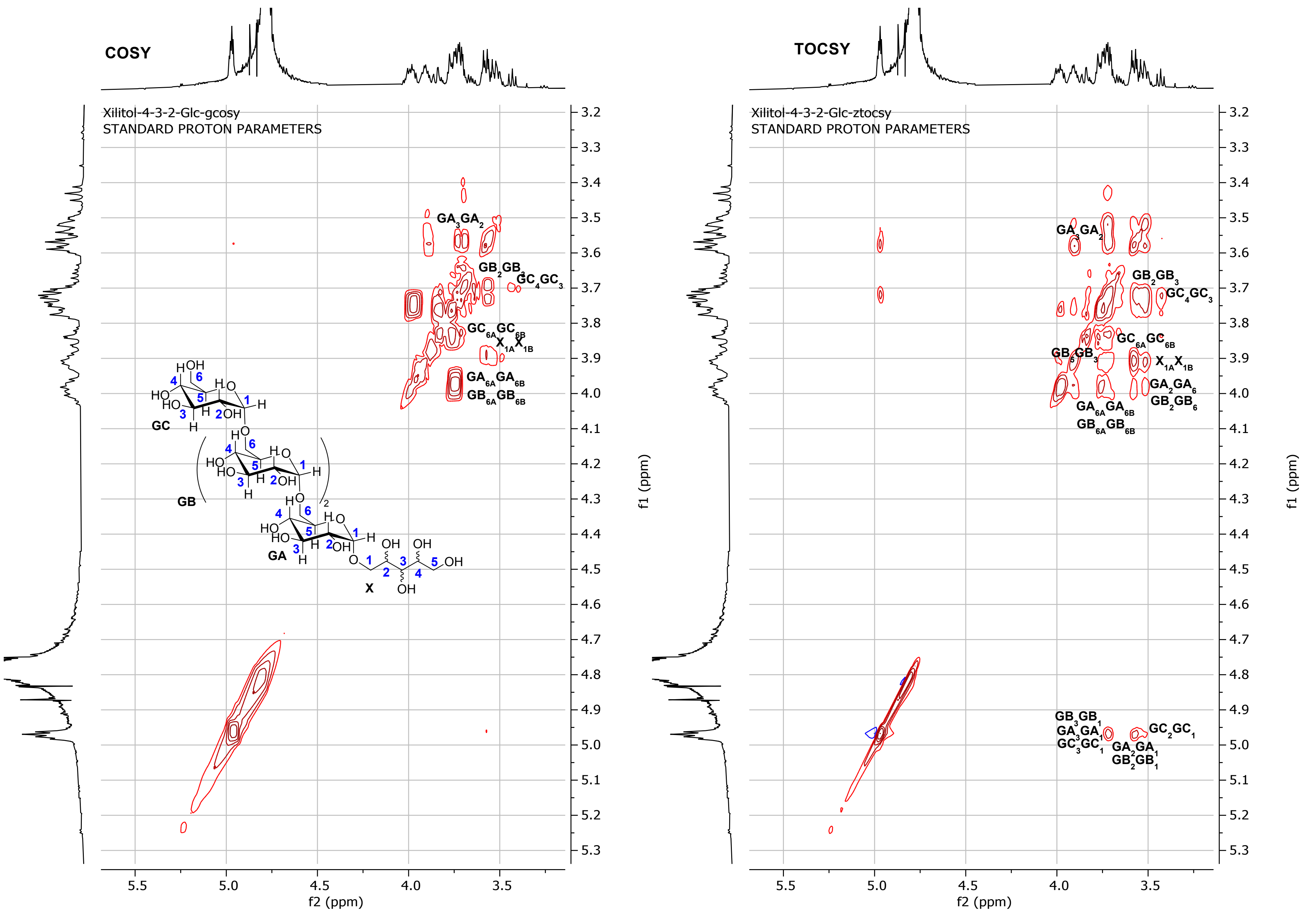

Figure S7. gCOSY and TOCSY (500 MHz, D_2O) of Xylitol-Glc.

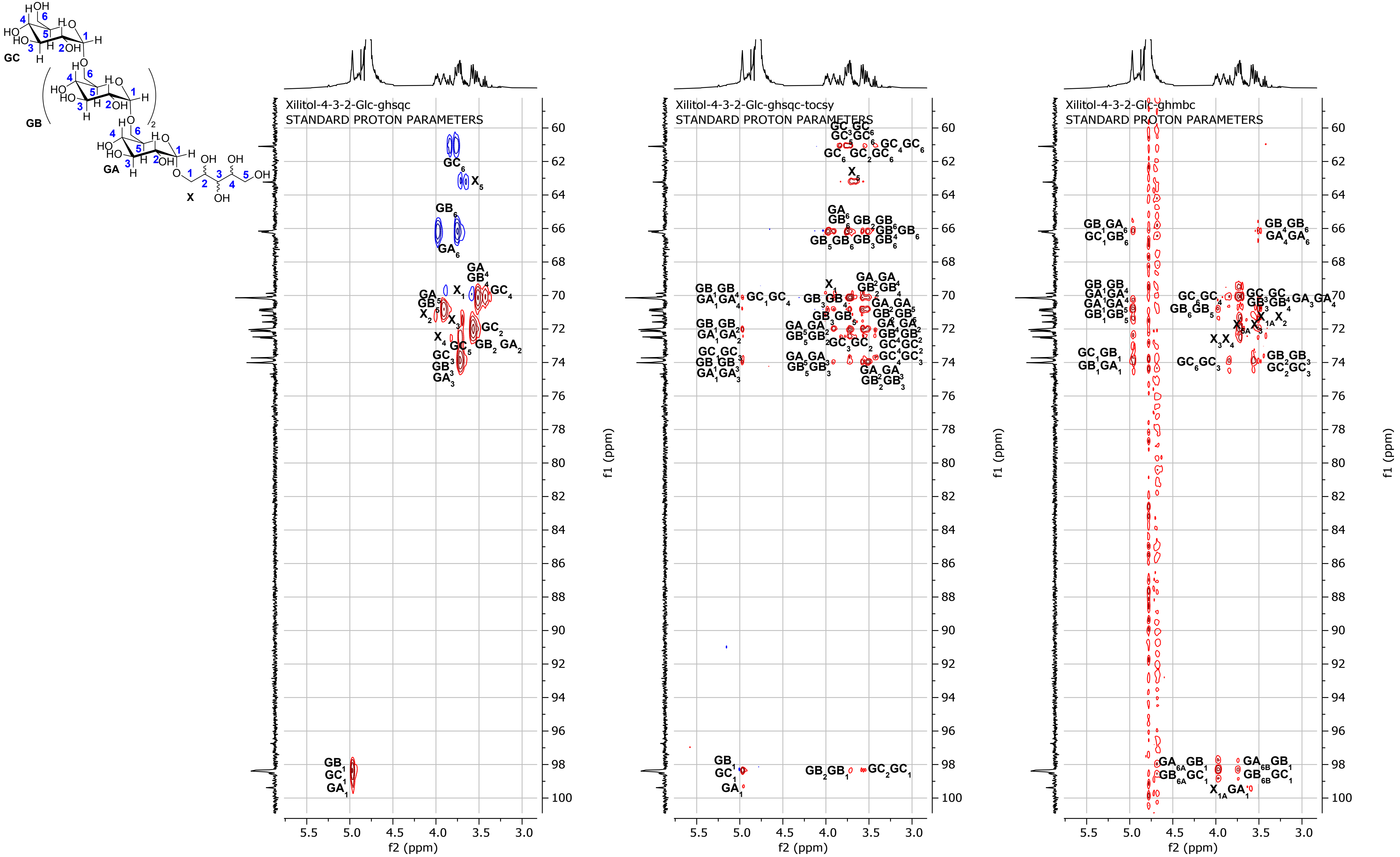

Figure S8. Multiplicity-edited gHSQC (methylene: blue cross peaks; methine: red cross peaks), gHSQC-TOCSY and gHMBC (500 MHz, D_2O) of Xylitol-Glc.

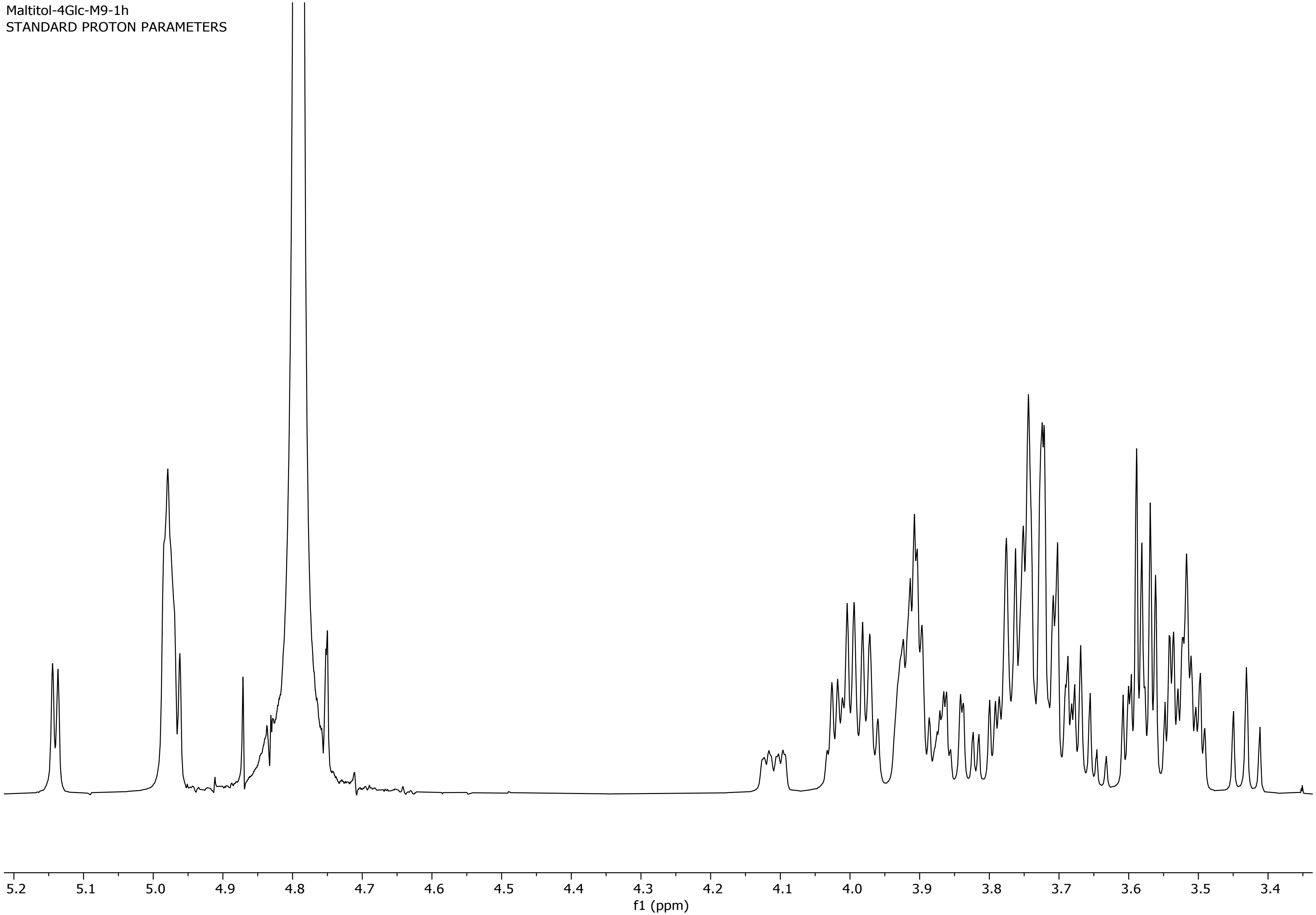

Figure S9. ^{1}H NMR (500 MHz, D_2O) of Maltitol-4Glc.

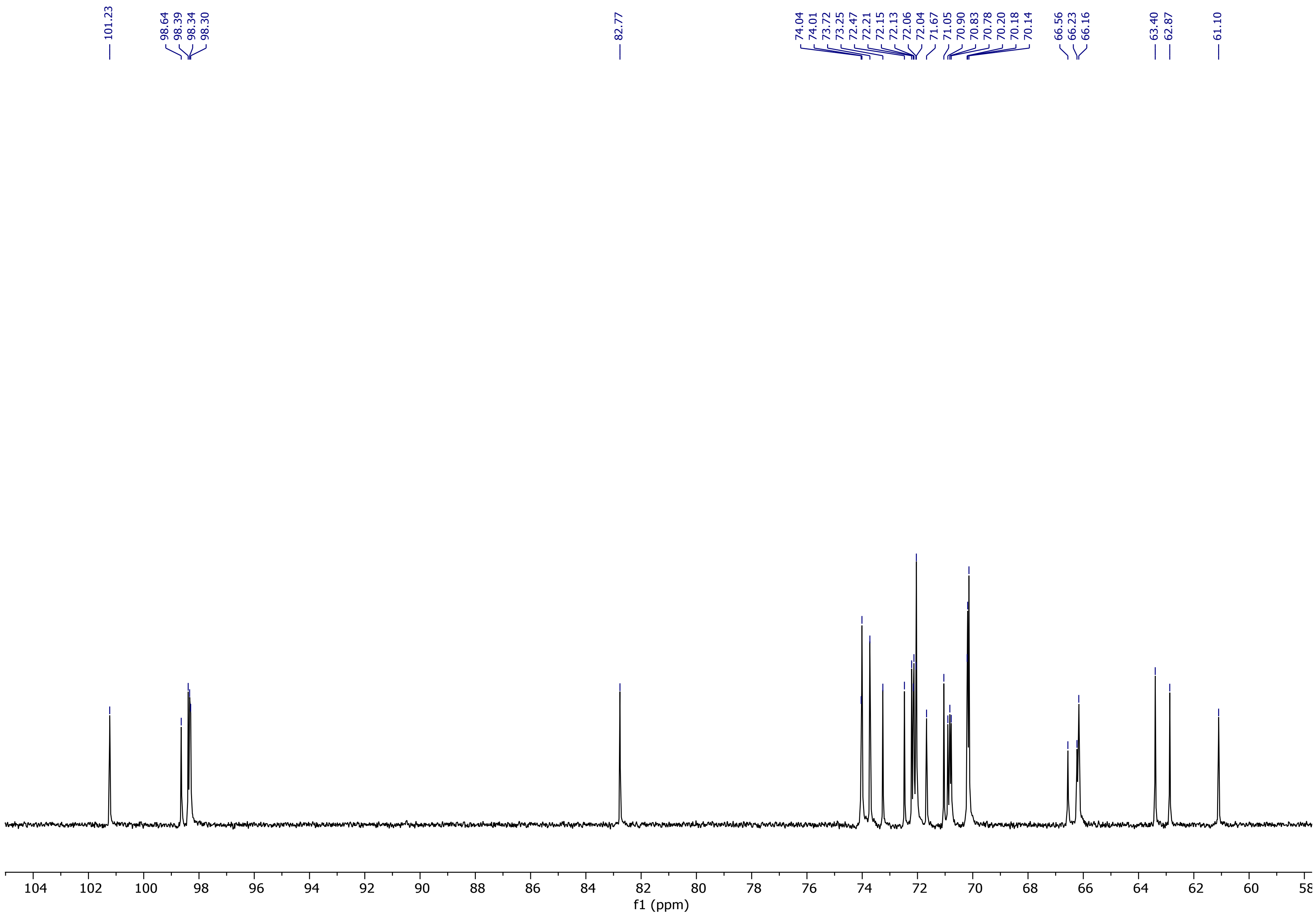

Figure S10. ^{13}C NMR (125 MHz, D_2O) of Maltitol-4Glc.

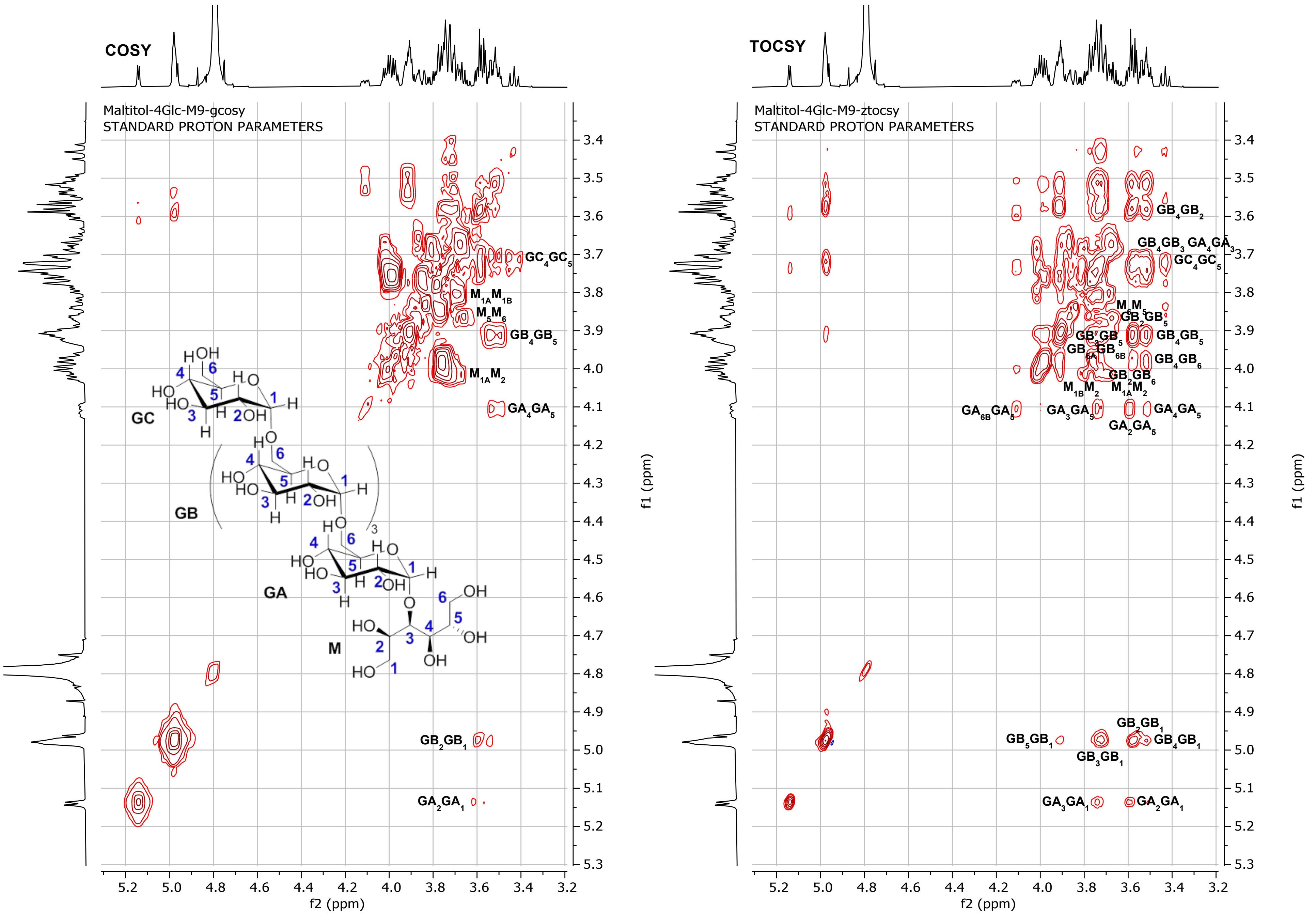

Figure S11. gCOSY and TOCSY (500 MHz, D_2O) of Maltitol-4Glc.

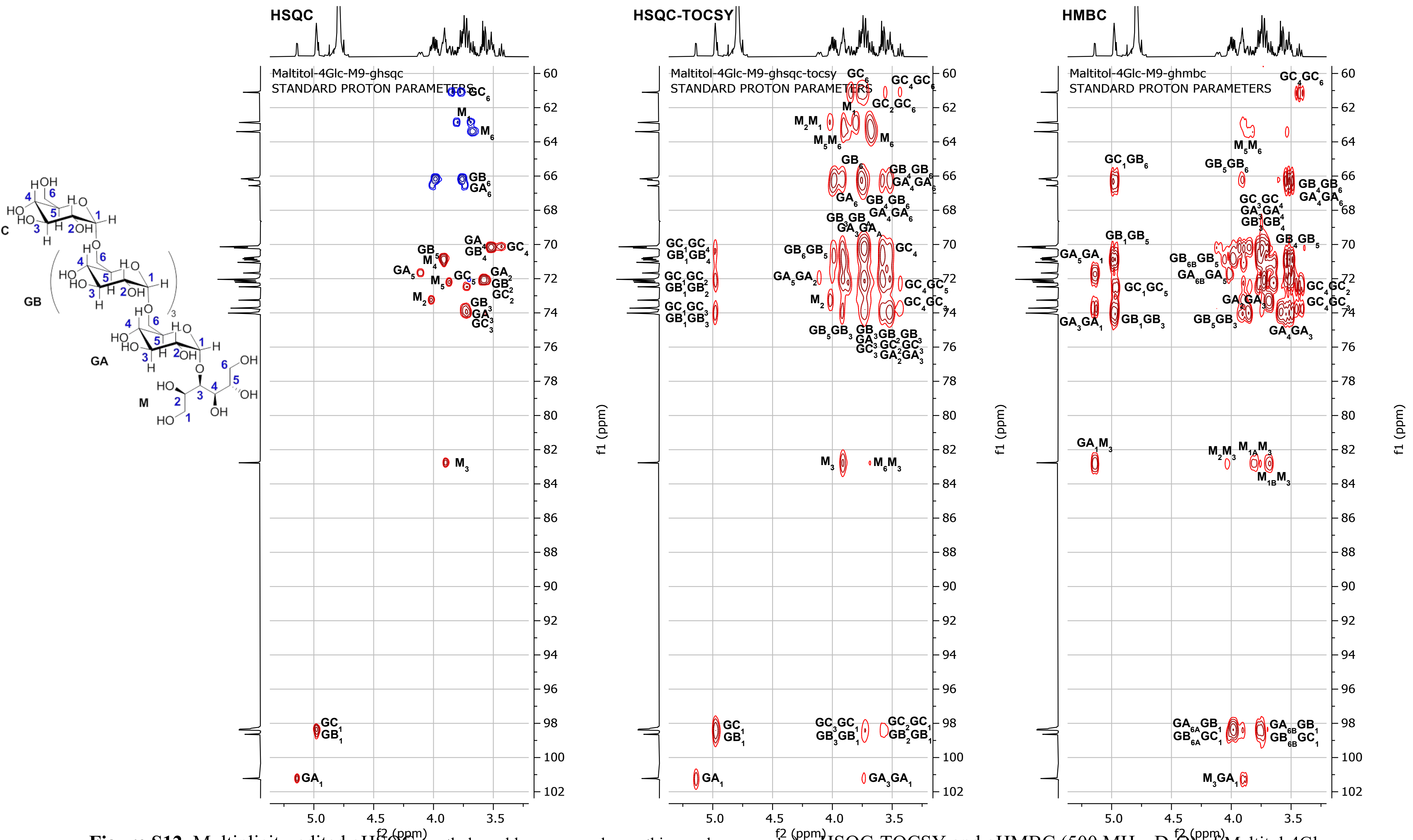

Figure S12. Multiplicity-edited gHSQC (methylene: blue cross peaks; methine: red cross peaks), gHSQC-TOCSY and gHMBC (500 MHz, D_2O) of Maltitol-4Glc.

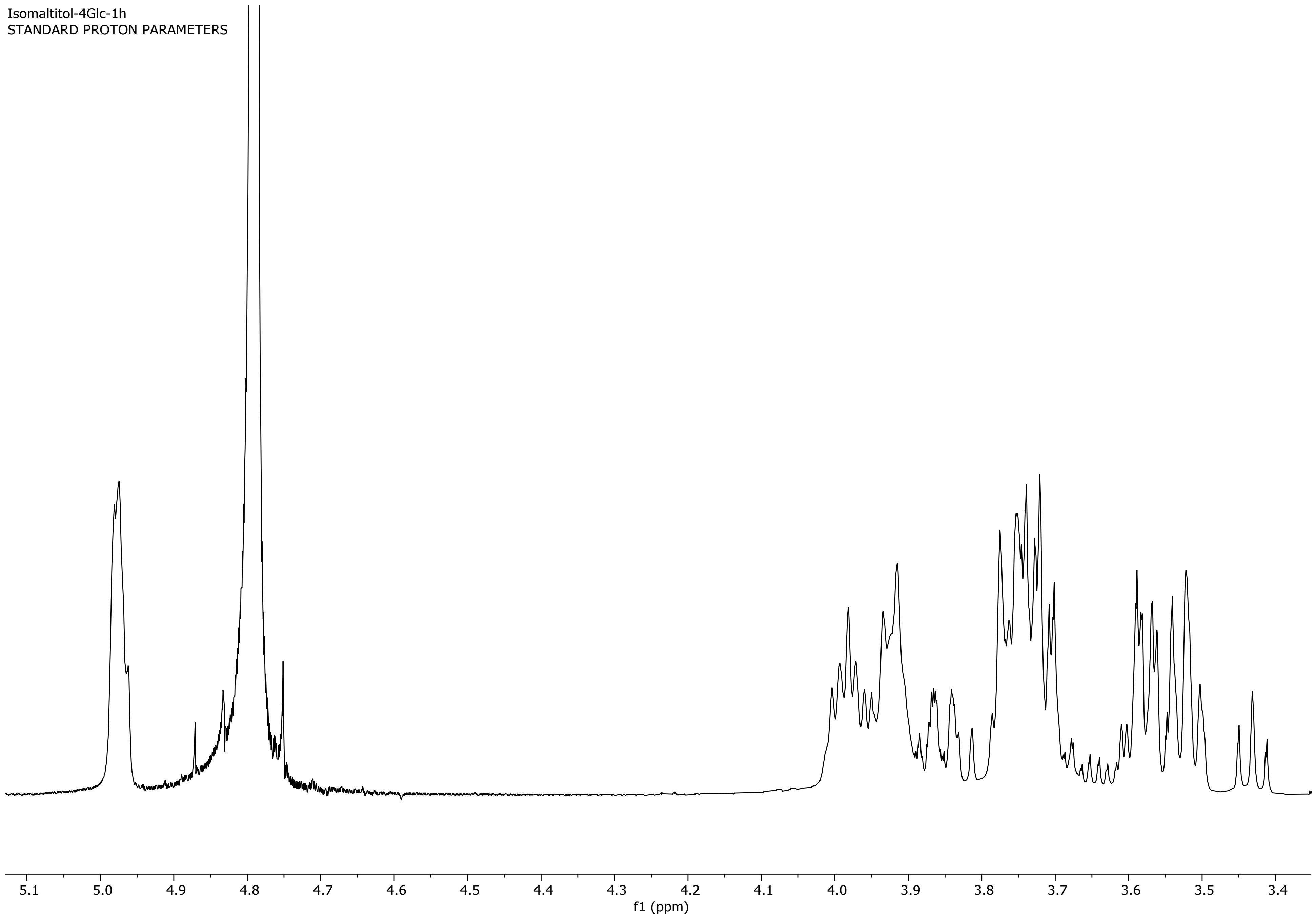

Figure S13. ^{1}H NMR (500 MHz, D_2O) of Isomaltitol-4Glc.

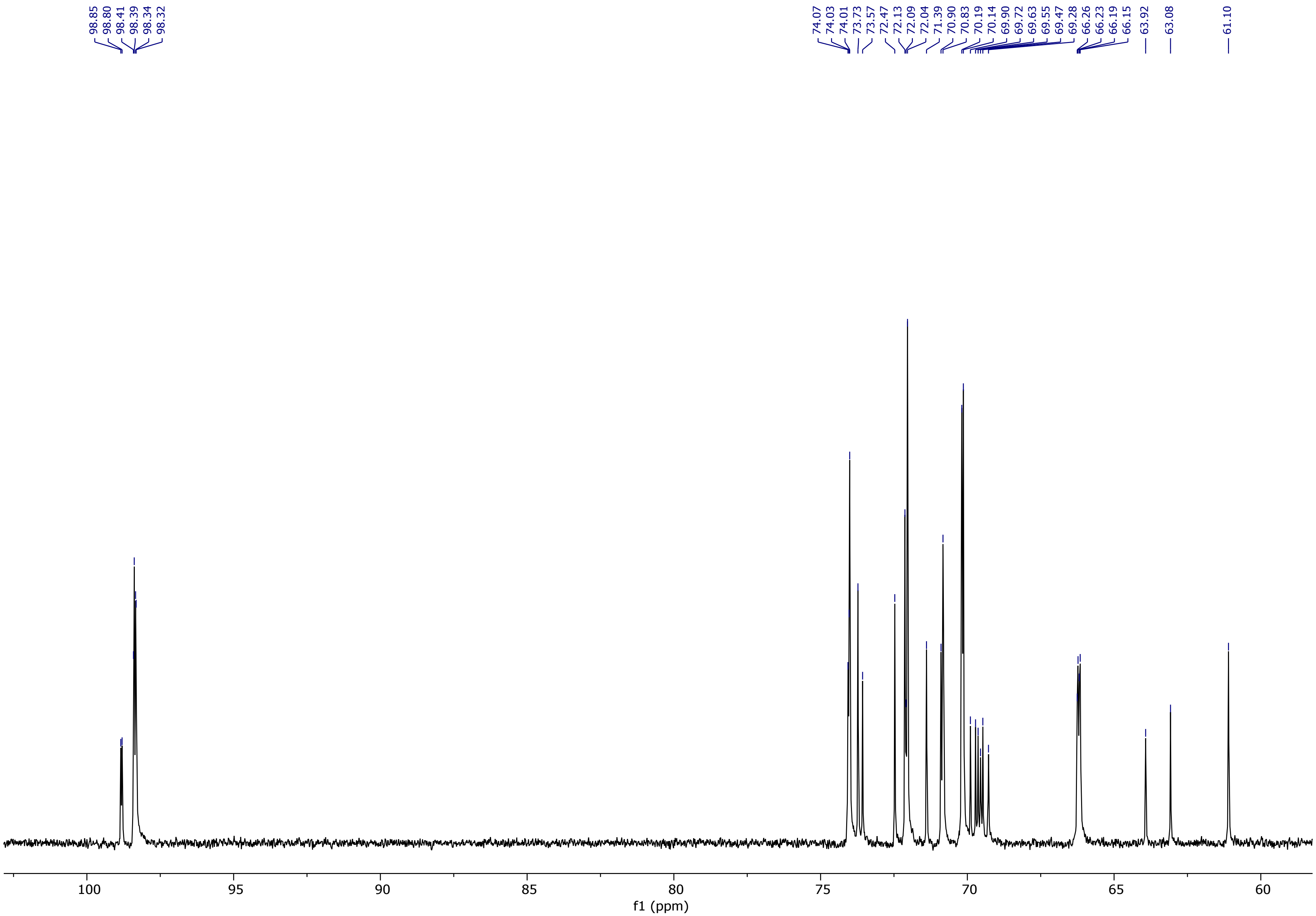

Figure S14. ^{13}C NMR (125 MHz, D_2O) of Isomaltitol-4Glc.

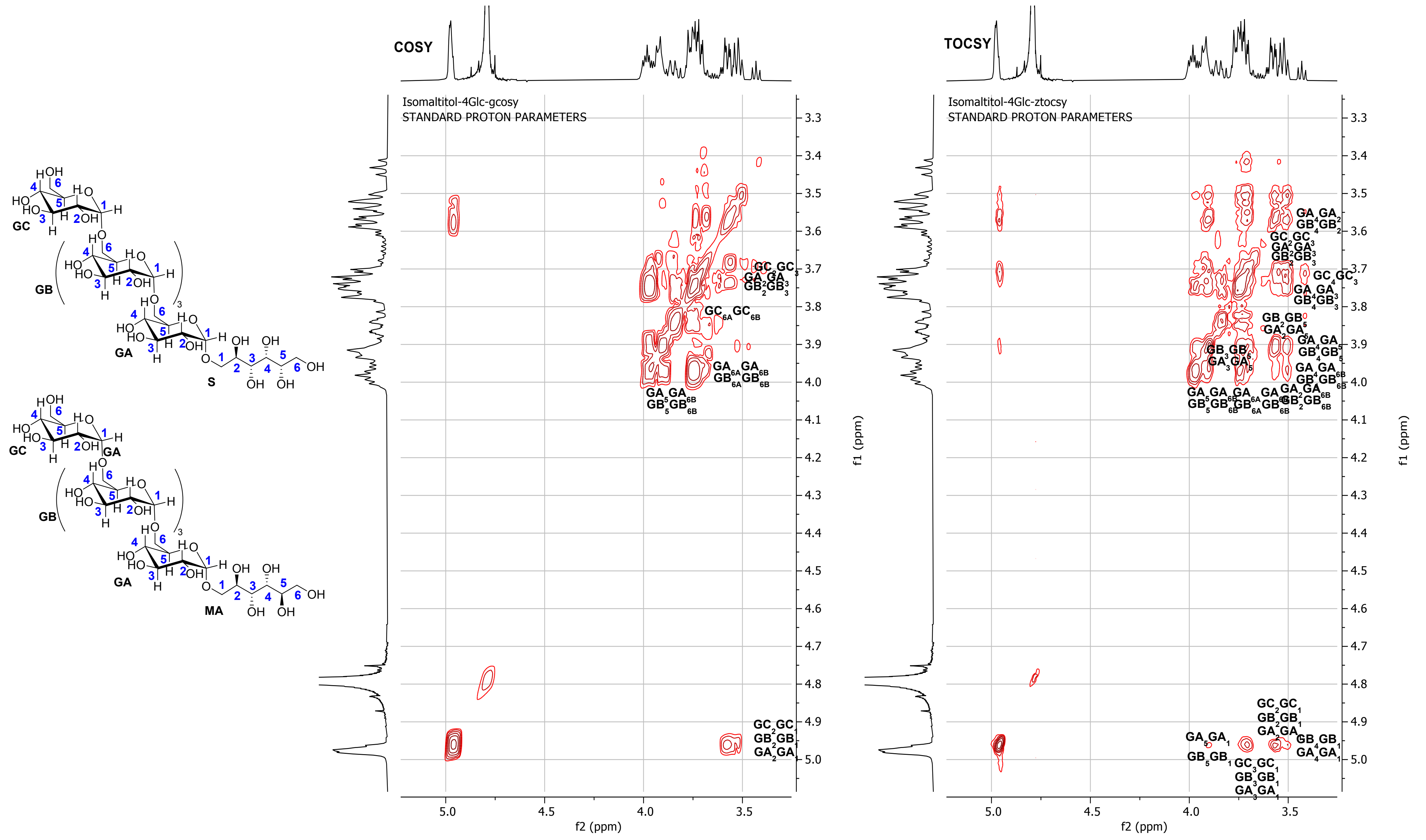

Figure S15. gCOSY and TOCSY (500 MHz, D_2O) of Isomaltitol-4Glc.

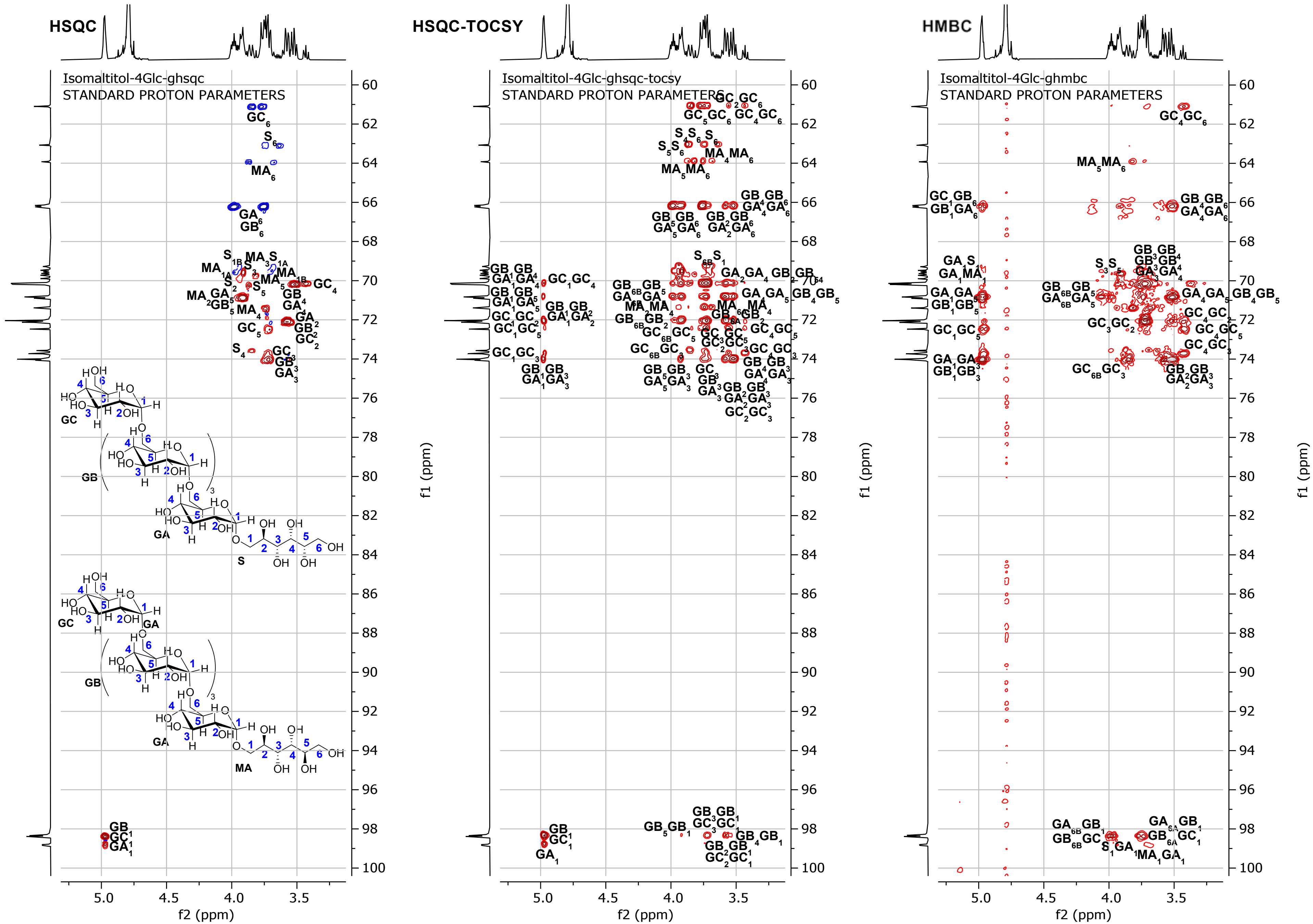

Figure S16. Multiplicity-edited gHSQC (methylene: blue cross peaks; methine: red cross peaks), gHSQC-TOCSY and gHMBC (500 MHz, D_2O) of Isomaltitol-4Glc.

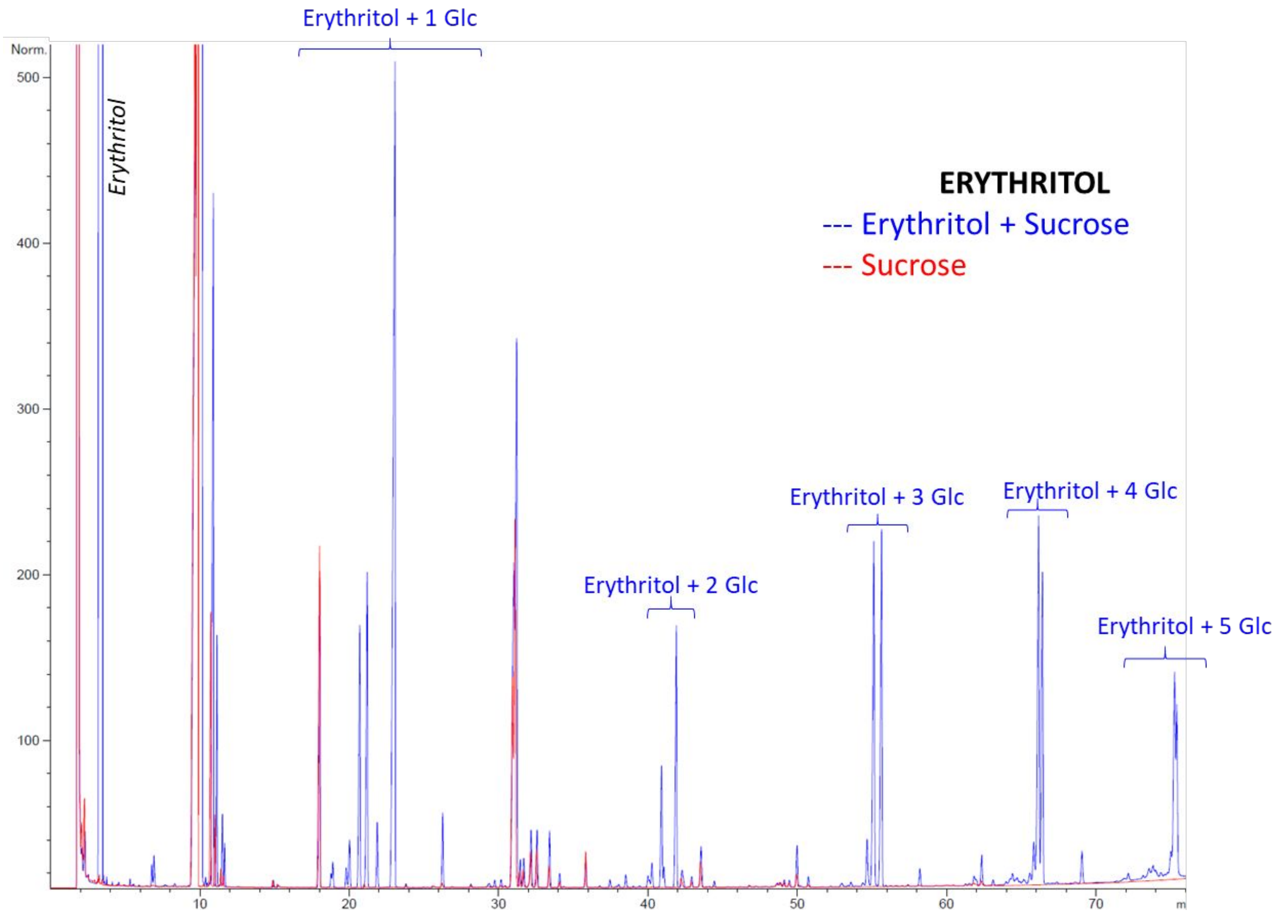

Figure S17. GC-FID profile of the enzymatic reaction with Dextransucrase using sucrose:erythritol as substrates.

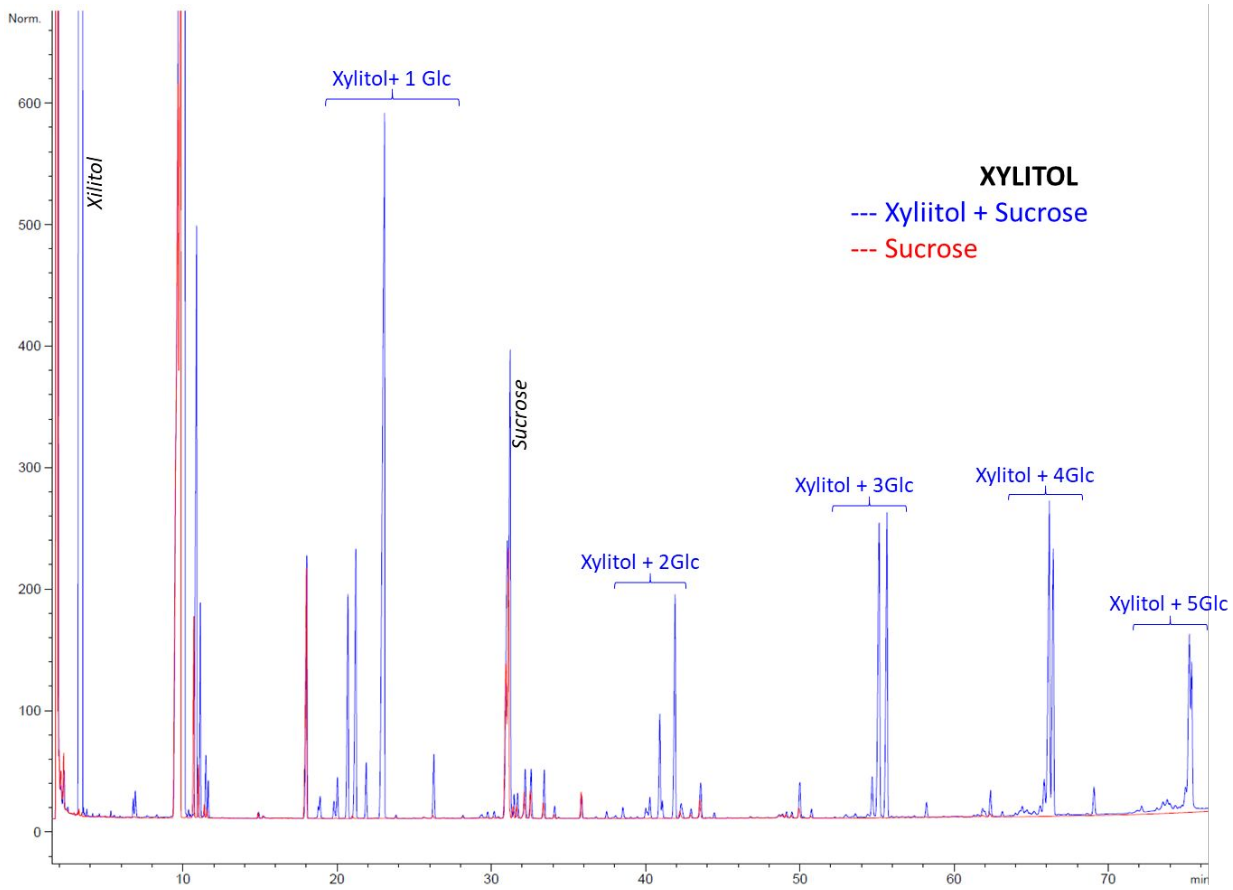

Figure S18. GC-FID profile of the enzymatic reaction with Dextransucrase using sucrose:xylitol as substrates.

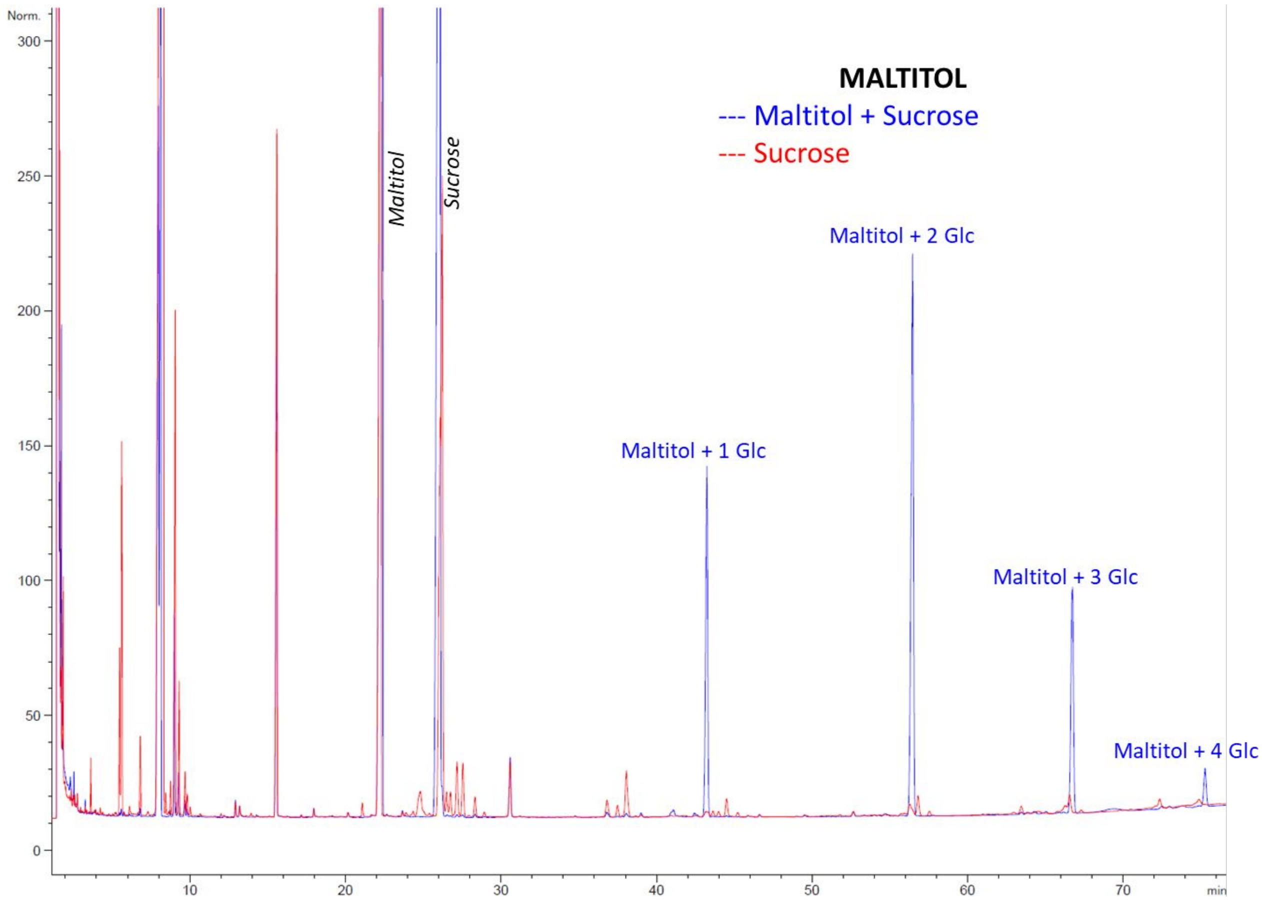

Figure S19. GC-FID profile of the enzymatic reaction with Dextransucrase using sucrose:maltitol as substrates.

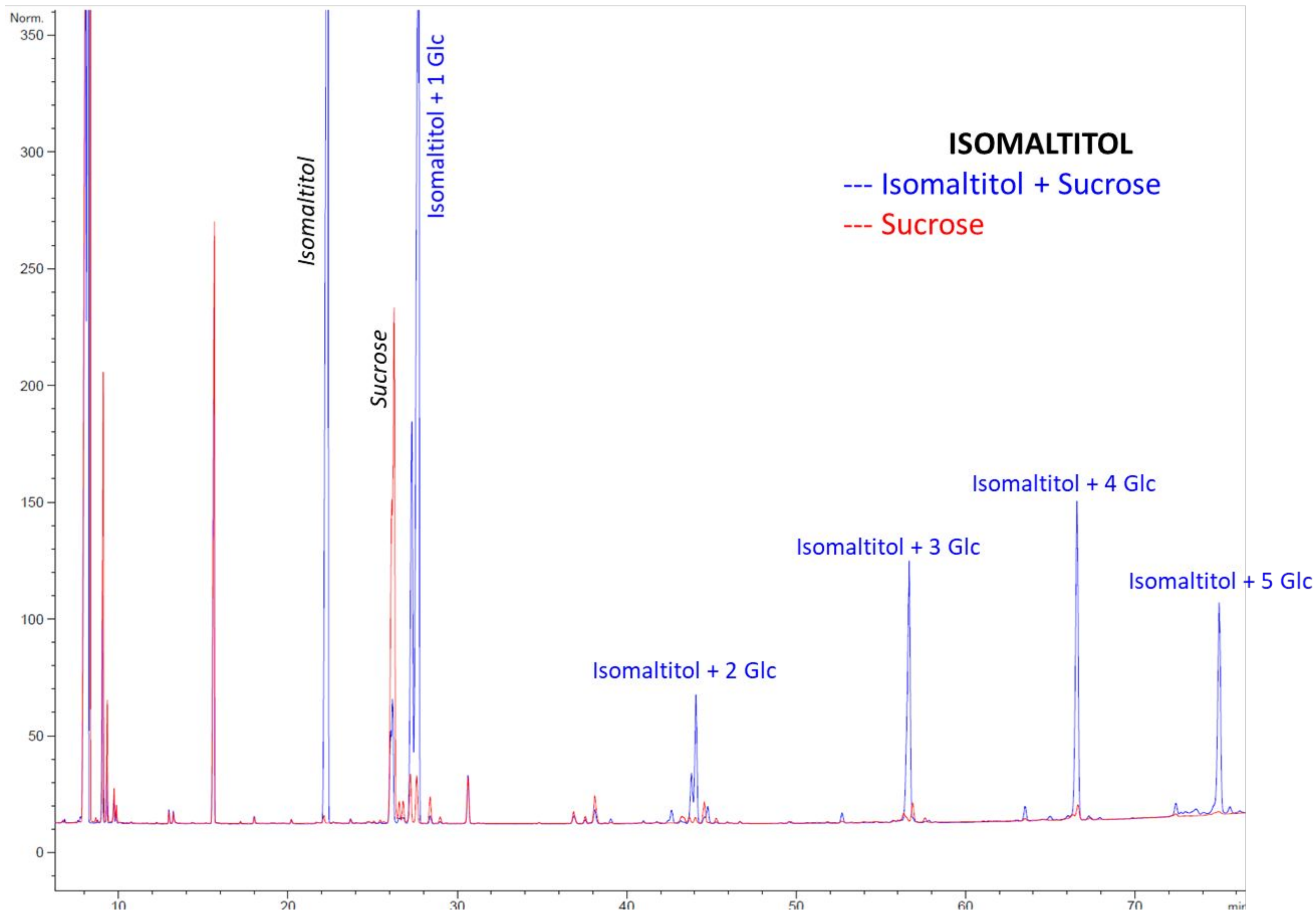

Figure S20. GC-FID profile of the enzymatic reaction with Dextransucrase using sucrose:isomaltitol as substrates.

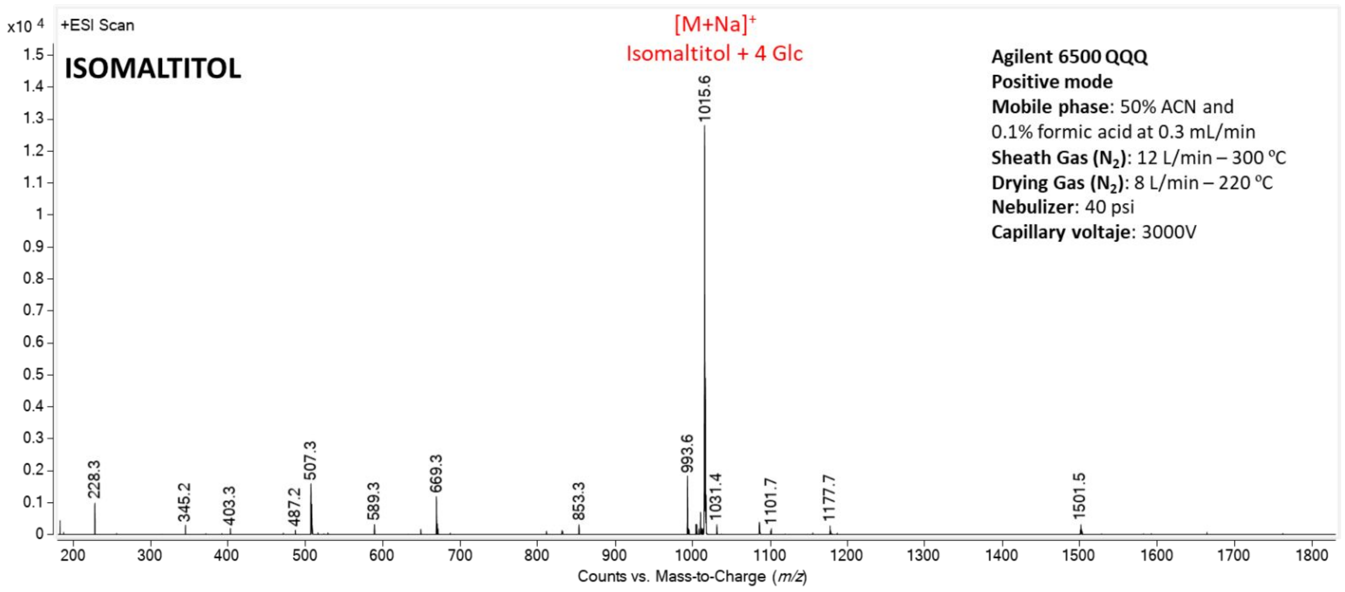

Figure S21. ESI-MS mass spectra of the selected fraction for isomaltitol purified by SEC and analysed by NMR.

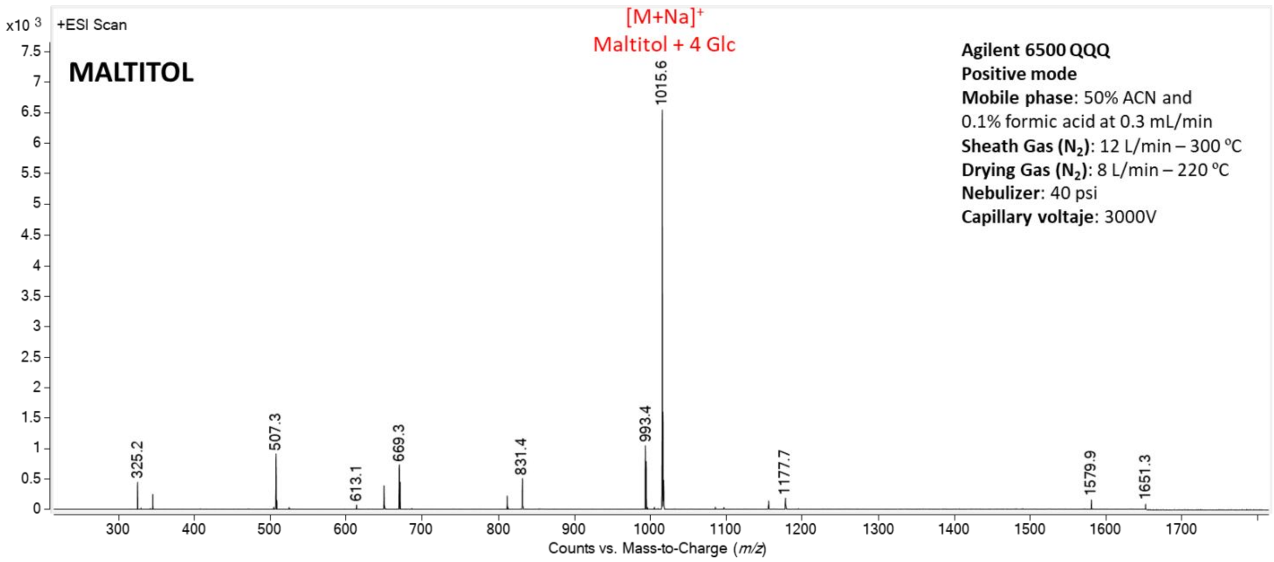

Figure S22. ESI-MS mass spectra of the selected fraction for maltitol purified by SEC and analysed by NMR.

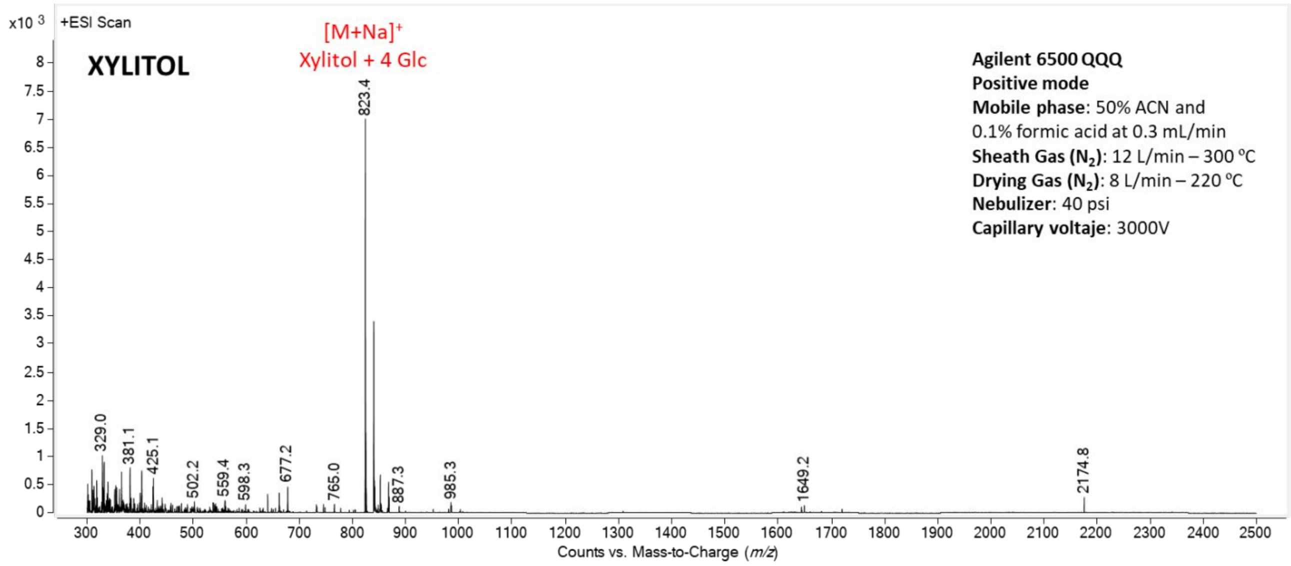

Figure S23. ESI-MS mass spectra of the selected fraction for xylitol purified by SEC and analysed by NMR.

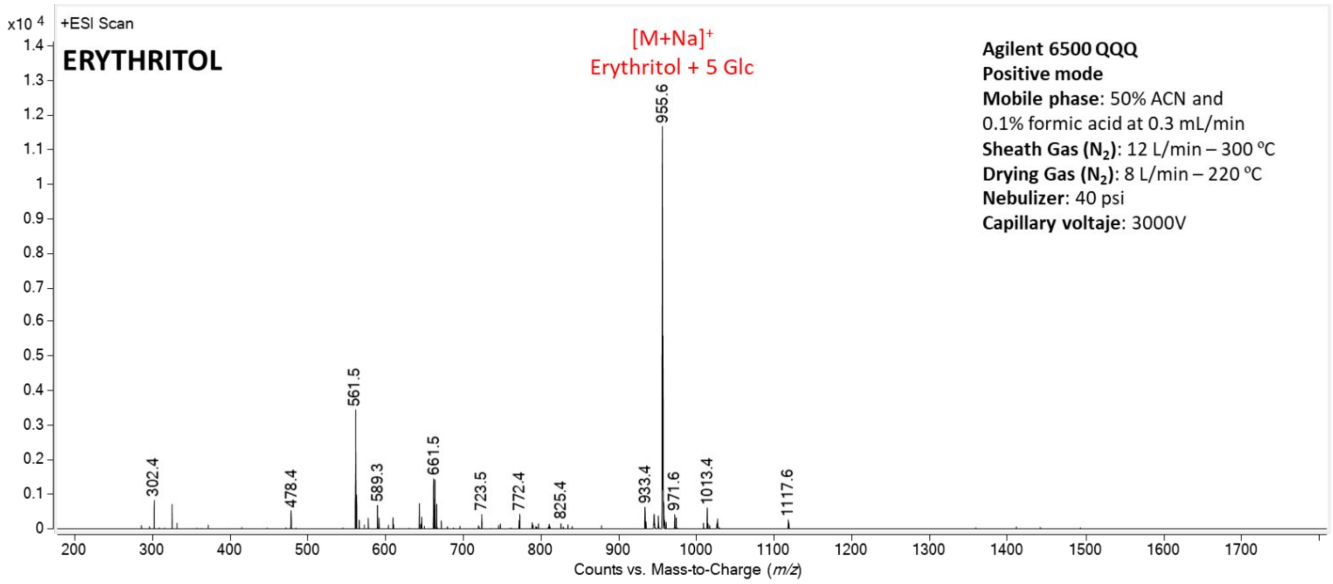

Figure S24. ESI-MS mass spectra of the selected fraction for erythritol purified by SEC and analysed by NMR.

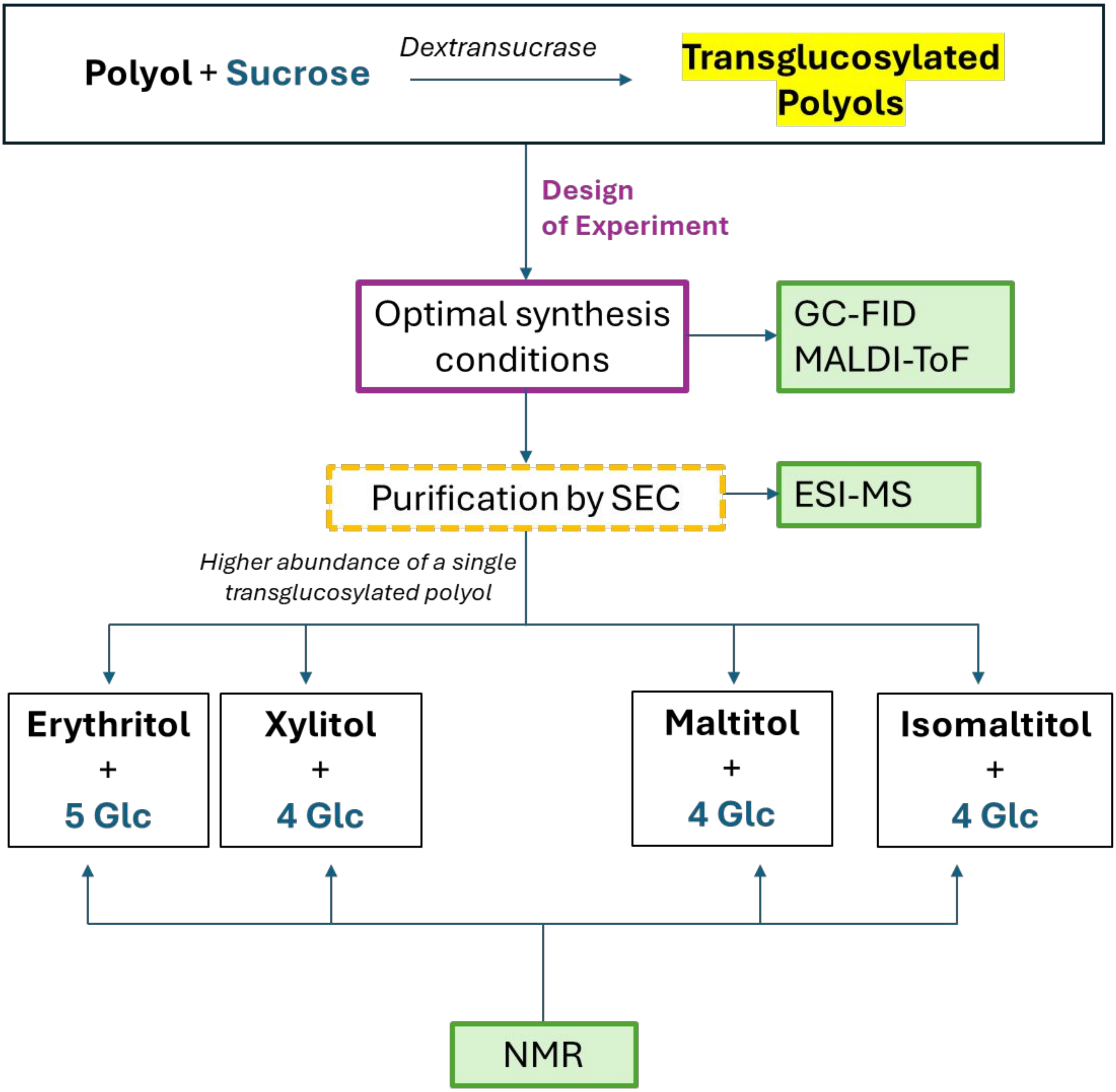

Figure S25. Flow scheme of the work process of the enzymatic reactions and the different techniques applied at the different stages.
